# The Benefit of Repairing the Deltoid Ligament in Unstable Ankle Fractures: Patient-Reported Functional Outcome and Radiological Stability Measurements; a Clinical Trial Protocol

**DOI:** 10.1177/24730114251386735

**Published:** 2025-11-12

**Authors:** Esten Konstad Haanæs, Frede Jon Frihagen, Greger Lønne, Aksel Paulsen, Jostein Skorpa Nilsen, Martin Greger Gregersen, Marius Molund

**Affiliations:** 1 Department of Neuromedicine and Movement Science, Norwegian University of Science and Technology, Trondheim, Norway; 2 Department of Orthopaedic Surgery, Østfold Hospital Trust, Grålum, Norway; 3 Department of Orthopaedic Surgery, Sykehuset Levanger, Helse Nord-Trondelag Hospital Trust, Levanger, Norway; 4 Institute of Clinical Medicine, University of Oslo, Oslo, Norway; 5 Department of Orthopaedic Surgery, Innlandet Hospital Trust, Tynset, Norway; 6 Department of Orthopaedic Surgery, Stavanger University Hospital, Stavanger, Norway; 7 Department of Public Health, Faculty of Health Sciences, University of Stavanger, Stavanger, Norway; 8 Department of Orthopaedic Surgery, Haukeland University Hospital, Bergen, Norway; 9 Department of Physical Medicine and Rehabilitation, Østfold Hospital Trust, Grålum, Norway; 10 Institute of Health and Society, Faculty of Medicine,University of Oslo, Oslo, Norway

**Keywords:** Outcome studies, sports, trauma, deltoid ligament, ankle fractures, weightbearing radiographs, posttraumatic arthritis, randomised controlled trial, research protocol

## Abstract

**Background:**

Suturing the deep posterior deltoid ligament in unstable ankle fractures is novel to established treatment. Some cadaveric and clinical trials support that adding deltoid ligament repair to plating of the lateral fracture will improve stability restoration.

**Objectives:**

We will investigate the effects of deep deltoid ligament repair on patient-reported function, radiologic stability parameters, and the incidence of ankle osteoarthritis and the possible side effects from this additional procedure. The medial ankle injury patterns found will be described.

**Study design:**

A randomised controlled nonblinded multicentre trial.

**Methods:**

A total of 120 patients with Lauge Hansen SER 4B ankle fractures will be randomised (1:1 ratio) to conventional plating of the lateral malleolus only or additional suture of the deep deltoid ligament. The primary end point was patient-reported function measured in Olerud-Molander Ankle Score (OMAS) at 1 and 2 years. The secondary end points included Self-Reported Foot and Ankle Score (SEFAS), Ankle Fracture Outcome of Rehabilitation Measure (A-FORM), VAS pain, and EuroQol-5D-5L scores; rates of treatment-related adverse events, reoperations, and incidence of posttraumatic arthritis; and comparison of side-to-side differences in tibiotalar medial clear space from bilateral weightbearing ankle radiographs and gravity stress on group level.

## Graphical abstract

**Graphical Abstract:**
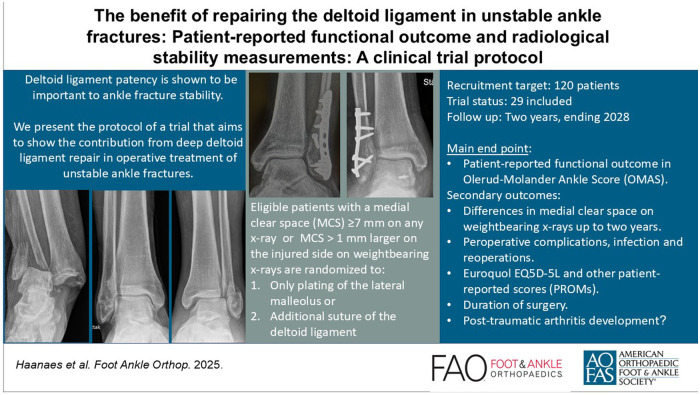

## Author Note

### Project Group: Participants, Organisation, and Collaborations

#### Steering group

*Project leader and co-supervisor:* Frede Frihagen, MD, PhD, consultant orthopaedic surgeon, ØHT and associate professor at OUS. Prof Frihagen has a vast research portfolio including planning and surveillance of international mulitcentre randomised controlled trials (RCTs), reviews for several orthopaedic journals, and leadership in international orthopaedic organisations.

*Main supervisor and principal investigator:* Marius Molund, MD, PhD, consultant orthopaedic surgeon, Department of Orthopaedic Surgery, ØHT. Marius has extensive clinical and research experience and is a reviewer of the journal *Foot and Ankle International*.

*PhD candidate:* Esten Konstad Haanæs, consultant orthopaedic surgeon, lead investigator at Sykehuset Levanger, and PhD candidate in ØHT and NTNU will be responsible for the day-to-day management of the RCT.

*Co-supervisors:*

Andrew M. Garratt, Dr. Scient, Norwegian Institute for Public Health (FHI). He has great experience in PROMs research and is the national main contact for EQ-5D and PROMIS.

Aksel Paulsen, MD, PhD, orthopaedic surgeon, head of research, associate professor, orthopaedic dept. Dr. Paulsen is lead investigator at Stavanger University Hospital and runs PROM research.

Greger Lønne,MD, PhD, consultant orthopaedic surgeon, Innlandet Hospital Trust (IHT), Tynset and associate professor, Norwegian University of Science and Technology (NTNU). NTNU has a data transfer agreement with Hemit, an information technology corporation that runs the research data base eFORSK. Hemit is owned by Central Norway Regional Health Authority. NTNU and ØHT are main institutions for the trial and have agreed upon shared responsibility for its data management.

*Lead investigators at other collaborating hospitals:*

Jostein Skorpa Nilsen, consultant orthopaedic surgeon, Haukeland University Hospital

Jakup Andreas Thomsen, consultant orthopaedic surgeon, Ålesund Sjukehus

Carl Erik Alm, consultant orthopaedic surgeon, Oslo University Hospital, Ullevål

Petter Grønmark, orthopaedic resident, ØHT

Henrik Emil Wildeng Pettersen, orthopaedic resident, SIHT, Gjøvik.

Ove Talsnes, consultant orthopaedic surgeon, SIHT, Elverum

Jonas Larsen Hilmo, Nordlandssykehuset Bodø

*Conflicts of interest:* We claim no relevant conflict of interest for the applicants.

## Introduction

Ankle fractures occur in up to 1 in 800 persons a year and are among the most frequent orthopaedic injuries.^28^ Post-traumatic arthritis, often causing pain and stiffness, is linked to the severity of the fracture and joint stability after treatment.^38,40^ Arthroplasty and arthrodesis in the ankle is by now less successful compared with arthroplasty in the hip and knee.^1^ Prevention by optimal fracture management will be preferable to treatment of established arthritis. The objective of this randomised controlled trial is the contribution from repair of the deep deltoid ligament in unstable Weber B ankle fractures. Because of increasing attention to deltoid ligament patency as fundamental to ankle joint stability, particularly in a fracture setting, of late, increasing interest has been paid to repairing the medial ankle ligament complex—both as an option or as an adjunct to osteosynthesis of the lateral malleolus and trans-syndesmotic fixation.

**Figure 1.**
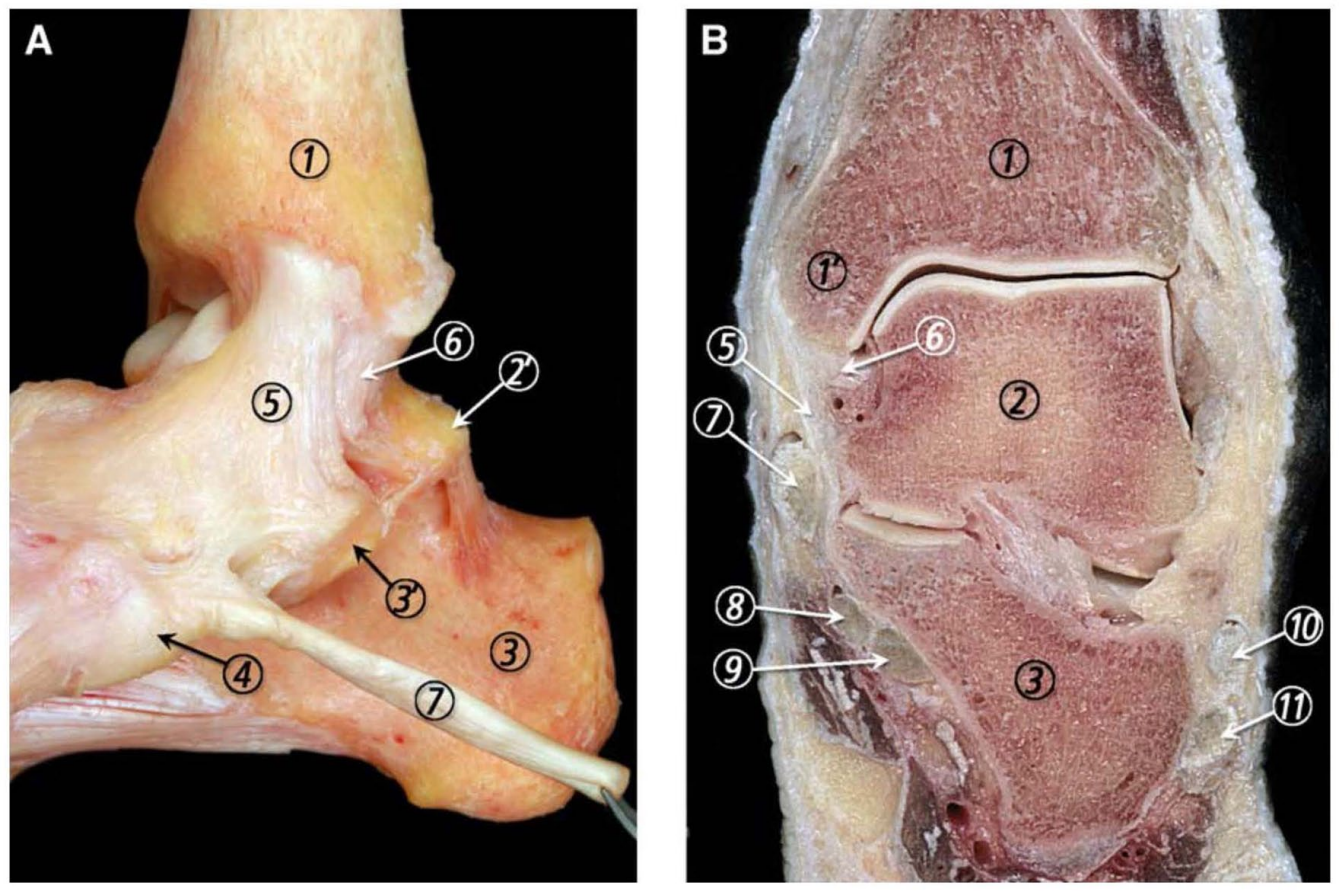
(A) Medial view of the ankle joint ligaments showing their typical fanlike morphology. (B) Frontal section of the ankle joint where the superficial and deep layers of the MCL (medial collateral ligament) are separated by a small mass of fatty tissue. 1, tibia; 1’, medial malleolus; 2, talus; 2’, medial talar process; 3, calcaneus; 3’, sustentaculum tali; 4, navicular tuberosity; 5, superficial layer of the MCL; 6, deep layer of the MCL; 7, tibialis posterior tendon; 8, flexor digitorum longus tendon; 9, flexor hallucis longus tendon; 10, peroneus brevis tendon; 11, peroneus longus tendon. Reprinted with permission from Elsevier^©^ (Golano et al^11^).

During the last few decades, less severe ankle fractures have been shown not to need operative treatment in general.^8,14^ As the total number of ankle fracture surgeries has decreased, treating the more complex fractures is still a challenge. Current surgical cases are more complex than in samples from the 3 recent decades, and the trend in treatment favours a more anatomical reconstruction. The use of weightbearing radiographs is now documented and established as a main tool in fracture stability assessment and guides the choice between conservative and operative treatment.^12,37,42^ Understanding these injuries implies recognising the role of the deep deltoid ligament as a main stabiliser of the ankle joint. (Figure 1).

Ankle fractures are traditionally classified by the anatomic region–based Weber-Danis or the aetiological Lauge-Hansen classification system.^20,41^ Unstable fibular fractures at the level of the ankle joint classify as Weber B/Lauge-Hansen supination external rotation 4b (SER4b). The stage SER4 subdivision into *a*, meaning partial injury, and *b*, complete deltoid ligament injury, is clinically important. Whether there is a complete deltoid ligament injury or not helps to separate fractures requiring operative treatment from the rest.^12^ Standard treatment for SER4b injuries has been plating of the distal fibula, screw fixation of the medial malleolus, and to a varying extent, syndesmotic fixation.^29^ If residual instability is found after fixation of the malleoli, the established surgical strategy has been to restabilise the distal tibiofibular joint. A trans-syndesmotic fixation, either by a screw or suture button, has been used most often, or when suitable, direct fixation of syndesmotic ligaments or avulsed syndesmosis-bearing fragments.^29^ Pakarinen et al^32^ were not able to show that SER4 fractures with a positive external rotation test after bony fixation did benefit from a trans-syndesmotic screw.

Cadaveric studies show that repair of the deep posterior deltoid ligament alone tends to give more stability than fixation of the lateral malleolus. Combining the two increases stability to a large extent.^3,7,13,29^ Some clinical studies suggest that deltoid ligament repair seems to have an effect on ankle mortise reduction and may prevent the development of posttraumatic arthritis.^18,35,38^ Deltoid ligament repair has been shown to give a more predictable reduction of the tibiofibular syndesmosis than performing a direct trans-syndesmotic fixation and considerably less frequent reoperations for hardware removal.^43,44^

Partial tears of the deltoid ligament are not rare in ankle fractures.^8,37^ Several of the patient samples in clinical reports on deltoid repair in ankle fractures are a mix of partial and complete ligament injuries. The way of repairing differs^21,22,33,38,45^ and is not always reported.^16,39^

Traditionally, repairing the deltoid ligament has been considered unnecessary, unless the ligament is interposed in the medial gutter and obstructing reduction of the ankle joint.^22^ The distal tibiofibular syndesmosis and deltoid ligament have a synergistic effect in ankle stability. When to fix the syndesmosis and when to suture the deltoid is not clear. As far as we know, no gold standard exists for operative treatment of SER4B fractures. The authors have experienced individual cases where deltoid ligament repair has been assessed to be necessary because of evident medial ankle instability after lateral fracture fixation. The effect of deep deltoid ligament repair in SER4b fractures and its effect on long-term function and arthritis is not yet known from larger clinical studies.

### Clinical Relevance

- A major number of fractures treated in former study samples were less severe and would be treated conservatively with current guidelines. This supports the need for studies on fractures still chosen for surgery with current guidelines.
- This trial is the first large and power-calculated RCT on deep deltoid ligament repair in Weber B SER4b fractures. It will evaluate whether additional ligament repair improves function compared with standard lateral fixation.
- Whether medial ankle joint congruency is reestablished and if deltoid ligament patency can be restored, will also be investigated, including whether this could help us avoid post-fracture arthritis.

## Methods

### Study Objectives

**Figure 2.**
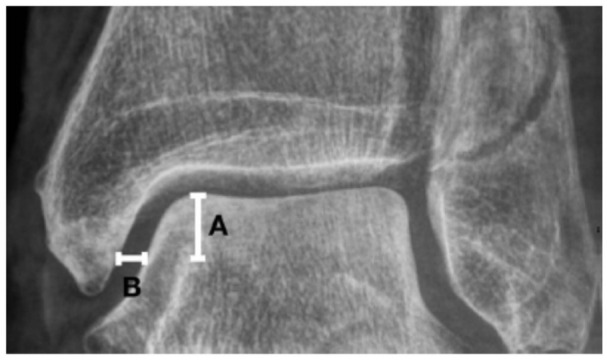
Method of obtaining medial clear space measurements from mortise view weightbearing radiographs. Line A is drawn from the top of the medial shoulder of the talar dome and 5.0 mm in the plantar direction. Medial clear space is measured as the distance between the talus and the medial malleolus on a line parallel to and 5.0 mm below the talar dome (line B).

**Figure 3.**
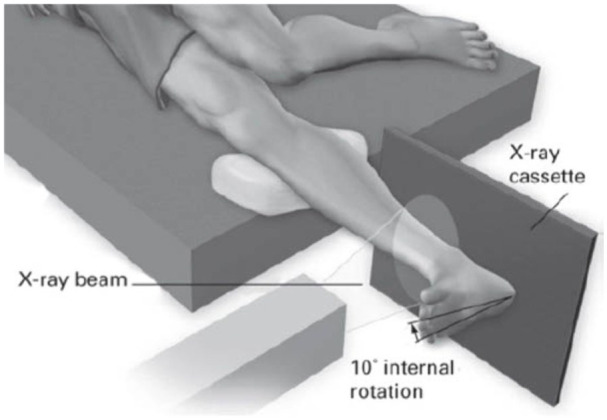
Gravity stress test after Schock et al JBJS(Br) 2007.^36^ Reprinted with permission from Springer^©^.

We hypothesize that functional outcome after additional deltoid ligament suture is clinically superior to that after solely plating the lateral malleolus in unstable Weber B fractures. We expect better joint reduction measured by MCS in millimetres on weightbearing radiographs, and less MCS increasement on gravity stress radiographs^14,27,36^ after deltoid ligament repair (Figures 2 and 3).

### Patient Collaboration

Patient board members from attending hospitals have contributed to the Norwegian translation and cross-cultural adaptation of the patient-reported outcome measures (PROMs) used and in the development of content and layout of the trial’s patient information.

### Trial Design

This is the research protocol for a preregistered ongoing multicentre randomised controlled trial with a clinical superiority design. The protocol is developed according to the SPIRIT 2025 statement.^4^ The trial has a pragmatic design.

### Study Setting

Østfold Hospital Trust (ØHT) is a public hospital in Norway and is responsible for the research trial, cooperating with the Norwegian University of Science and Technology (NTNU). Both local, regional, and national trauma hospitals are recruiting to the trial. All 4 health regions of Norway are represented. The trial’s catchment area serves more than 2 000 000 inhabitants. Patients arriving at recruiting hospitals receive oral and written information about the trial from the orthopaedic resident surgeon on call or a physiotherapist or nurse in the emergency department or outpatient clinic. It will be stressed that participation is voluntary, and that they can withdraw their consent at any time without influencing their further treatment.

### Choice of Comparators

We have chosen lateral plating only as comparator and additional deltoid ligament repair as intervention. Different options have been discussed before choosing treatment arms, also simply deltoid ligament repair without plating of the lateral malleolus as an intervention, to turn a Lauge-Hansen SER4b into an SER2 fracture, a fracture that could be treated without surgery.^20^ This suggestion was found to be too controversial.

We know that a trans-syndesmotic fixation has a synergistic effect to deltoid ligament repair in stabilising the ankle fork around the talus, and probably holding the torn deltoid ligament ends in proximity in most cases. We see the risk of trans-syndesmotic fixations hiding the potential effect of deep posterior deltoid ligament repair.

Nor do we know the effect of trans-syndesmotic fixation in a sample of SER4B fractures. This could have been a third arm of treatment. At the same time, we know that trans-syndesmotic fixations carry a high malreduction rate and the need for implant removal is quite frequent.^9^ When we were about to start the trial, realizing that established practice differs in the attending hospitals, we could not defend prohibiting the use of trans-syndesmotic implants in either group. The chosen arms of treatment attempts to be a pragmatic approach where the main difference between the groups will be whether the deep posterior deltoid ligament is repaired or not. We acknowledge that the solely plating treatment arm may contain several trans-syndesmotic fixations that may compensate for and conceal the true benefit from deltoid ligament repair compared with plating only.

### Interventions

Operative treatment should be performed within 2 weeks after injury. Treatment in both arms of randomisation must be performed by equally experienced surgeons, a consultant or fellowship-trained orthopaedic surgeon taking part in the procedure, preferably a foot and ankle surgeon, or experienced trauma surgeon. Preoperative CT should be performed as a general routine in cases of suspicion of additional injuries.

### Surgical technique

#### Both treatment arms

The lateral malleolus shall be fixed with a plate respecting the principles of modern fracture treatment. Particularly in cases with several fragments or expecting inferior bone density, we recommend anatomic plates with angular stability. After lateral plating, further testing is optional, that is, talar shift and tilt or for syndesmotic instability.

#### Concomitant care

If the surgeon notices severe instability that he or she finds compulsory to address further, this should be performed, and additional fixation will be registered.

#### Patients randomised to additional deltoid ligament repair

Deltoid ligament repair is done by a curved incision following the path of the tibialis posterior tendon from just proximal to the posterior limit of the medial malleolus till past the anterior tip. The tendon retinaculum is incised in the direction of the tendon.

**Figure 4.**
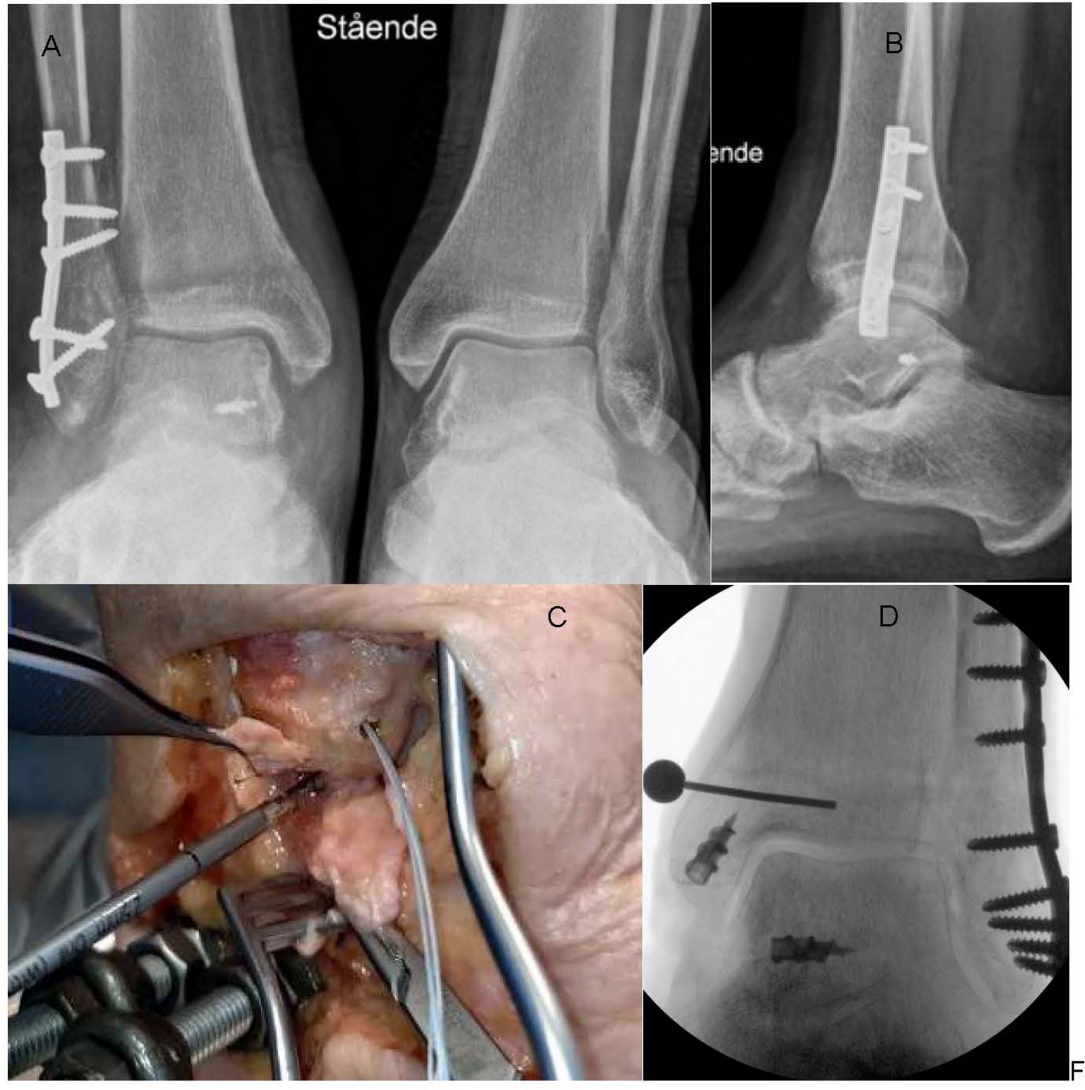
Deltoid ligament repair examples. (A) Weightbearing X-ray of operatively treated SER4B fracture. Suture anchor in the talus just plantar to the medial malleolus holds the deep posterior deltoid ligament parts. (B) Note the short distance from the suture anchor to the subtalar joint. (C) Cadaveric model of deltoid ligament repair showing a suture anchor placed in the colliculus anterior of the medial malleolus. The needle holder retracts anterior parts of the deltoid ligament. Drilling in the medial side of the talus, just beneath the colliculus posterior, for attachment of the anchor for posterior ligament parts being lifted by the forceps. (D) X-ray from a cadaveric study showing anchor placement in a cadaveric SER4B fracture model, also stabilized by a lateral anatomic plate.

-Note the degree of injury to the deep posterior deltoid ligament and site of tear (talar or tibial end or midsubstance).
-Because the deep posterior part most often is torn on its talar end,^17^ the routine repair will be suturing its fibres to a bone anchor in the talus. We recommend temporary pin placement and fluoroscopy in at least 1 projection (anteroposterior view) to confirm correct positioning before anchor placement. A modern bone anchor must be used (Figure 4). We recommend using a knot pusher to achieve a firm repair as the knot will be hard to get to just beneath the posterior medial malleolus.
-Another suture anchor in the anterior colliculus of the medial malleolus is optional.
-We close more superficial and anterior parts of the ligament tear before suturing the tibialis posterior-retinaculum, which is closed with a resorbable No. 1 suture or stronger.
-We suggest medial ligament repair prior to lateral plating to get a better overview.

#### Aftertreatment

Postsurgery, up to 2 weeks, the patient is in a cast or uses a walker boot when loading a maximum of 20 kg. After 2 weeks, free movement with or without a walker boot or similar is allowed, with weightbearing as tolerated.

### Randomisation

Eligible patients are allocated in a 1:1 ratio to either solely plating of the lateral fracture or additional deltoid ligament repair. Block randomisation is stratified for biological sex and age <50 or ≥50 years at the time of inclusion. Eligibility criteria are stated in Table 1. The enrolment procedure is described in the SPIRIT 2025 Schedule (Table 2).^4^

**Table 1.** Eligibility Criteria.

Patients are eligible if they present to one of the participating hospitals and comply with the inclusion and exclusion criteria.	
Inclusion Criteria	Exclusion Criteria
18–65 y of age at presentation	Assumed not compliant (eg, drug use, cognitive and/or psychiatric disorders)
Initial medial clear space (MCS) ≥7 mm or weightbearing radiograph (WBXR) evaluated as unstable (side-to-side difference >1 mm or fracture dislocation; when in doubt, WBXR to be performed)	Insufficient language skills (Scandinavian)
Able to walk without aids before the injury	Multitrauma or pathologic fracture
Posterior malleolus fragment Mason and Molloy 1 or no posterior malleolus fragment^15^	Neuropathies and symptomatic generalised joint disease (eg, rheumatoid arthritis)
Surgery planned within 2 wk after injury and available for follow-up	Previous ipsilateral ankle surgery or fracture, or previous injury with marked sequela of the lower limb
No syndesmotic screw or suture button planned before the surgical procedureNo other more severe condition in the same extremity	Open fracture Gustilo-Anderson II or more^23^ or other medial soft tissue problem considerably increasing risk of additional medial approach to the ankle.

**Table 2.** SPIRIT 2025 Diagram of the Schedule of Enrolment, Interventions, and Assessments.

Time point				
Enrolment	Screening for eligibility	See eligibility criteria (Table 1).		
	Informed consent	Written and oral information from a physiotherapist, nurse, or orthopaedic surgeon other than the operating surgeon		
	Randomisation	Randomisation module in the eFORSK database (block randomisation)		
Surgical intervention (within 2 wk after injury)	Intervention: Additional deltoid ligament repair	Surgical treatment will be performed by equally experienced surgeons in both the intervention and comparator groups: Fellowship-trained foot and ankle or trauma surgeons with lower extremity experience. Observations from hospital stay and operation: injury and repair description, duration of surgery; pre- and perioperative complications, length of hospital admission		
	Comparator: Plating of the lateral malleolus only			
Rehabilitation protocol	Equal for both study arms	Up to 2 weeks in cast or walker boot and partial weightbearing 20 kg; thereafter movement exercises and weightbearing as tolerated; orthosis optional from 2 wk		
Assessments				
Time of follow-up	WBXR^a^	Gravity Stress XR	PROMs (E-survey)	Other
6 wk ±10 d	X			VAS pain, adverse events, use of orthosis and supporting aids
12 wk ± 2 wk	X		X	As for 6 wk, time of return to work
1 y ± 1 mo	X	X	X^b^	Validation of test-retest properties
2 y ± 2 mo	X		X	Last observation for main publication
5 y ± 5 mo	X	X	X	Will be published in a later paper

Abbreviations: PROMs, patient-reported outcome measures; VAS, visual analogue scale; WBXR, weightbearing radiography; XR, radiography. a) Weight-bearing x-rays (WBXR) of both legs in the mortise projection and measurement of MCS at all times of follow-up. b) PROMS are sent twice for test-retest validation of the PROMs used in Norwegian versions, in particular the Ankle Fracture Outcome of Rehabilitation Measure (A-FORM) translation.

### Concealment and Blinding

As no sham surgery will be performed to the medial side of the ankle in the osteosynthesis-only treatment arm, and suture anchors often may be seen on radiographs, blinding will not be possible. Operating surgeons follow up their own patients.

### Outcomes

#### Primary outcome

Patient-reported function in Olerud-Molander Ankle Score (OMAS) at 1 and 2 years. Results at 12 weeks will also be reported. Times of assessment are described in Table 2.

#### Secondary outcomes

Infection, reoperation, and other major adverse events shall be registered and compared between groups.

- Function specific to the anatomic region reported by Ankle Fracture Outcome of Rehabilitation Measure (A-FORM), Self-Reported Foot and Ankle Score (SEFAS),^5,6,10,26,31^ and visual analogue scale (VAS) pain.
- EuroQoL–5 Dimensions, 5 Levels (EQ-5D-5L) (general health status).^34^
- Difference in medial clear space on weightbearing radiographs or gravity stress images after surgery with or without deltoid ligament repair.
- Signs of postfracture arthritis is another important outcome, and it will be reported according to the Kellgren-Lawrence scale.^2,19^

The phenomenon of *ceiling effect* means high score in most patients answering a PROM.^24^ This has been a challenge in the long-term follow-up of former studies on ankle fracture patients. When selecting PROMs as end points for our study, we have looked for PROMs asking about the ability to fulfil difficult tasks to reach the highest score range.

### Sample Size and Power Calculations

To detect a defined minimal clinically important difference (MCID) of 9.7 in OMAS between the study groups, expected to be between 8 and 10, we chose 8. In addition, we chose an SD of 14. Different reported SDs are 13 and 15 in the groups of Pakarinen et al^32^ and Molund et al^30^ had an SD of 12 on average. With our defined significance level *α* = 0.05, Power 0.8, we estimated almost 50 patients in each group. The calculation was done by a 2-sided *t* test, clinical superiority design. We will include 60 patients in each group to ensure sufficient power after expected drop-out.^46^ The trial is underpowered to expect significant results from its subgroups.

### Data Management and Storage

All data will be entered and stored electronically in the electronic research database (eFORSK, Hemit, Trondheim, Norway). Access to the study data is restricted to study group members, and the hospitals’ lead investigators access their own hospitals’ data through the national health network by 2-factor authentication. eFORSK holds a computerised randomisation module. The research database also collects PROM data electronically from the patients at follow-up. Data will be deidentified before export for statistical analysis. Participant files will be maintained in storage for a period of 5 years after the completion of the study before deletion. Deidentified individual participant data will be shared on reasonable request.

### Statistical Analysis

Descriptive data will be presented as means with SDs, medians with range, or frequencies and percentages when appropriate. The nonparametric Mann-Whitney *U* test will be used to compare groups where data are skewed, for instance, for PROM scores. A 2-sided *t* test will be used to analyse differences for normally distributed data. Categorical data will be analysed using the Fisher exact test. We will conduct logarithmic transformation and linear regression or if more appropriate quartile regression, for multivariate regression using the generalised estimating equation model. Data will be analysed using the IBM SPSS Statistics and Stata. The significance level is set to 5%. Comparisons will also be done between different groups of stratification, and the 25% of patients having the worst outcome in each treatment arm. All statistical analyses will be performed in cooperation with the statisticians at ØHT and NTNU. Reasons for ineligibility, noncompliance, withdrawal, or other protocol violations will be stated, and any patterns will be summarised. A more detailed statistical plan is available as Supplementary file 2.

### Monitoring and Harms Reporting Procedures

Complications will be discussed among local investigators and the steering group in search for avoidable causes and best possible follow-up. Actions to prevent further adverse events will be made. If several occur recruitment may be stopped locally or in general. Major adverse events will be reported promptly to our Data Safety and Monitoring Board (DSMB), which have a mandate to stop the study if untoward effects are observed. All adverse events will be reported every 6 months, and preliminary results from the first 30 patients’ 12 weeks follow-up presented. Interim analysis will be performed of PROM scores and adverse events for the first 60 patients at the 1-year follow-up.

## Trial Status

When submitting this protocol, 29 patients out of our target of 120 (24%) are enrolled in the study. Recruitment began in September 2024, and we anticipate ending within the winter of 2026-2027.

## Ethics and Dissemination

Deltoid ligament repair might be challenging for surgeons with limited specific ankle surgery experience. The protocol requires participation of an experienced trauma surgeon or a foot and ankle surgeon. We do not find deltoid ligament repair difficult to teach. We believe the moderate risk implied by this ligament suture may be outweighed by improved talar reduction, ankle stability, and function. Surgeons picked to perform the procedures in collaborating hospitals have been offered teaching and cadaveric procedure training. Some side effects from deltoid ligament repair like medial ankle stiffness or pain from the tibialis posterior tendon and prolonged procedure time may be expected.

Results will be communicated to attending patients and clinicians, presented in regional and national media and published in peer-reviewed journals. We will also be presenting at national and international conferences.

The Regional Committee for Medical and Health Research Ethics (REK Sørøst, ref. 496556) and the Data Protection Officers at all collaborating hospitals have approved patient recruitment to this trial. Participation is based on consent. Patient board members have contributed to the development of patient information. Data collection and management follow approval terms.

The trial is preregistered as NCT 2024104 at ClinicalTrials.gov.

## Supplemental Material

sj-docx-4-fao-10.1177_24730114251386735 – Supplemental material for The Benefit of Repairing the Deltoid Ligament in Unstable Ankle Fractures: Patient-Reported Functional Outcome and Radiological Stability Measurements; a Clinical Trial Protocol Supplemental material, sj-docx-4-fao-10.1177_24730114251386735 for The Benefit of Repairing the Deltoid Ligament in Unstable Ankle Fractures: Patient-Reported Functional Outcome and Radiological Stability Measurements; a Clinical Trial Protocol by Esten Konstad Haanæs, Frede Jon Frihagen, Greger Lønne, Aksel Paulsen, Jostein Skorpa Nilsen, Martin Greger Gregersen and Marius Molund in Foot & Ankle Orthopaedics

sj-docx-5-fao-10.1177_24730114251386735 – Supplemental material for The Benefit of Repairing the Deltoid Ligament in Unstable Ankle Fractures: Patient-Reported Functional Outcome and Radiological Stability Measurements; a Clinical Trial Protocol Supplemental material, sj-docx-5-fao-10.1177_24730114251386735 for The Benefit of Repairing the Deltoid Ligament in Unstable Ankle Fractures: Patient-Reported Functional Outcome and Radiological Stability Measurements; a Clinical Trial Protocol by Esten Konstad Haanæs, Frede Jon Frihagen, Greger Lønne, Aksel Paulsen, Jostein Skorpa Nilsen, Martin Greger Gregersen and Marius Molund in Foot & Ankle Orthopaedics

sj-docx-6-fao-10.1177_24730114251386735 – Supplemental material for The Benefit of Repairing the Deltoid Ligament in Unstable Ankle Fractures: Patient-Reported Functional Outcome and Radiological Stability Measurements; a Clinical Trial Protocol Supplemental material, sj-docx-6-fao-10.1177_24730114251386735 for The Benefit of Repairing the Deltoid Ligament in Unstable Ankle Fractures: Patient-Reported Functional Outcome and Radiological Stability Measurements; a Clinical Trial Protocol by Esten Konstad Haanæs, Frede Jon Frihagen, Greger Lønne, Aksel Paulsen, Jostein Skorpa Nilsen, Martin Greger Gregersen and Marius Molund in Foot & Ankle Orthopaedics

sj-pdf-1-fao-10.1177_24730114251386735 – Supplemental material for The Benefit of Repairing the Deltoid Ligament in Unstable Ankle Fractures: Patient-Reported Functional Outcome and Radiological Stability Measurements; a Clinical Trial Protocol Supplemental material, sj-pdf-1-fao-10.1177_24730114251386735 for The Benefit of Repairing the Deltoid Ligament in Unstable Ankle Fractures: Patient-Reported Functional Outcome and Radiological Stability Measurements; a Clinical Trial Protocol by Esten Konstad Haanæs, Frede Jon Frihagen, Greger Lønne, Aksel Paulsen, Jostein Skorpa Nilsen, Martin Greger Gregersen and Marius Molund in Foot & Ankle Orthopaedics

sj-pdf-2-fao-10.1177_24730114251386735 – Supplemental material for The Benefit of Repairing the Deltoid Ligament in Unstable Ankle Fractures: Patient-Reported Functional Outcome and Radiological Stability Measurements; a Clinical Trial Protocol Supplemental material, sj-pdf-2-fao-10.1177_24730114251386735 for The Benefit of Repairing the Deltoid Ligament in Unstable Ankle Fractures: Patient-Reported Functional Outcome and Radiological Stability Measurements; a Clinical Trial Protocol by Esten Konstad Haanæs, Frede Jon Frihagen, Greger Lønne, Aksel Paulsen, Jostein Skorpa Nilsen, Martin Greger Gregersen and Marius Molund in Foot & Ankle Orthopaedics

sj-pdf-3-fao-10.1177_24730114251386735 – Supplemental material for The Benefit of Repairing the Deltoid Ligament in Unstable Ankle Fractures: Patient-Reported Functional Outcome and Radiological Stability Measurements; a Clinical Trial Protocol Supplemental material, sj-pdf-3-fao-10.1177_24730114251386735 for The Benefit of Repairing the Deltoid Ligament in Unstable Ankle Fractures: Patient-Reported Functional Outcome and Radiological Stability Measurements; a Clinical Trial Protocol by Esten Konstad Haanæs, Frede Jon Frihagen, Greger Lønne, Aksel Paulsen, Jostein Skorpa Nilsen, Martin Greger Gregersen and Marius Molund in Foot & Ankle Orthopaedics

sj-pdf-7-fao-10.1177_24730114251386735 – Supplemental material for The Benefit of Repairing the Deltoid Ligament in Unstable Ankle Fractures: Patient-Reported Functional Outcome and Radiological Stability Measurements; a Clinical Trial Protocol Supplemental material, sj-pdf-7-fao-10.1177_24730114251386735 for The Benefit of Repairing the Deltoid Ligament in Unstable Ankle Fractures: Patient-Reported Functional Outcome and Radiological Stability Measurements; a Clinical Trial Protocol by Esten Konstad Haanæs, Frede Jon Frihagen, Greger Lønne, Aksel Paulsen, Jostein Skorpa Nilsen, Martin Greger Gregersen and Marius Molund in Foot & Ankle Orthopaedics
