# Supplementary material for: The Benefit of Repairing the Deltoid Ligament in Unstable Ankle Fractures: Patient-Reported Functional Outcome and Radiological Stability Measurements; a Clinical Trial Protocol
Source: Foot Ankle Orthop. 2025 Nov 12;10(4):24730114251386735. doi: 10.1177/24730114251386735 (PMC12615956; doi:10.1177/24730114251386735)
Supplement: sj-docx-4-fao-10.1177_24730114251386735 – Supplemental material for The Benefit of Repairing the Deltoid Ligament in Unstable Ankle Fractures: Patient-Reported Functional Outcome and Radiological Stability Measurements; a Clinical Trial Protocol [file sj-docx-4-fao-10.1177_24730114251386735.docx]

**Table 1 | SPIRIT 2025 checklist of items to address in a randomised trial protocol**

| **Section/topic** | **No** | **SPIRIT 2025 checklist item description** |
| --- | --- | --- |
| **Administrative information** | | |
| Title and structured summary | 1a | The benefit of repairing the deltoid ligament in unstable ankle fractures, a multicentre randomized controlled study. Patient-reported functional outcome and radiological stability measurements. |
|  | 1b | South-Eastern Norway Regional Health Authority project number ***2024104***  Date of first enrolment was 30^th^ of September 2024.  Recruitment status: Recruiting.  Completion date: 1^st^ of July 2032.  Country of recruitment: Norway  ***Contact for public queries:***  Esten Konstad Øiaas Haanæs  Consultant orthopaedic surgeon and PhD candidate  Norwegian University of Science and Technology (NTNU)  Helse Nord-Trøndelag Hospital Trust, Østfold Hospital Trust and Norwegian Armed Forces  Box 333  N-7601 Levanger  [estenkh@gmail.com](mailto:estenkh@gmail.com)  Phone: +4799789013  ***Contact for scientific queries:***  Principal investigator: Prof. Dr. Med Frede Jon Frihagen  [Frede.Frihagen@so-hf.no](mailto:Frede.Frihagen@so-hf.no)  Sicentific contact: Dr. Med. Marius Molund, Head of Department of Orthopaedic Surgery  [marius.molund@so-hf.no](mailto:marius.molund@so-hf.no)  Department of Orthopaedic surgery. Østfold Hospital Trust.  Kalnesveien, Box 300  N-1714 Grålum  Phone: +47 69 86 00 00 |
| Protocol version | 2 | 2nd version, 23^rd^ of january 2025, publishing version |
| Roles and responsibilities | 3a | Names, affiliations and roles of protocol contributors  **Participants, organization and collaborations**  **Steering group:**  Project leader and co-supervisor: Frede Frihagen, MD, PhD, consultant orthopaedic surgeon, Østfold Hospital Trust (ØHT) and associate professor at Oslo University Hospital.  Main supervisor and principal investigator: Marius Molund, MD, PhD, consultant orthopaedic surgeon, Department of orthopaedic surgery, ØHT. Marius has extensive clinical and research experience and is a reviewer of the journal Foot and Ankle International.  PhD candidate: Esten Konstad Haanæs, consultant orthopaedic surgeon, Lead investigator at Sykehuset Levanger and PhD candidate in ØHT, will be responsible for the day-to-day management of the RCT.  Co-supervisors: Andrew M. Garratt, Dr. Scient, Norwegian Institute for Public Health (FHI). He has great experience in PROMs research and is the national main contact for EQ-5D and PROMIS.  Aksel Paulsen, MD, PhD, orthopaedic surgeon, head of research, associate professor, orthopaedic dept. Dr. Paulsen is lead investigator at Stavanger University Hospital and runs PROMs research.  Greger Lønne,MD, PhD, consultant orthopaedic surgeon, Innlandet Hospital Trust (IHT), Tynset and associate professor, Norwegian University of Science and Technology (NTNU). NTNU has a data transfer agreement with Hemit, an information technology corporation that runs the research data base eFORSK. Hemit is owned by Central Norway Regional Health Authority. NTNU and ØHT are main institutions for the trial and have agreed upon shared responsibility for its data management.  Lead investigators at other collaborating hospitals:  Aleksander Uchermann and Jostein Skorpa Nilsen, consultant orthopaedic surgeons, Haukeland University Hospital  Jakup Andreas Thomsen, consultant orthopaedic surgeon, Ålesund Sjukehus, Helse Møre og Romsdal Hospital Trust  Carl Erik Alm, consultant orthopaedic surgeon, Oslo University Hospital, Ullevål  Petter Grønmark, orthopaedic resident, ØHT  Henrik Emil Wildeng Pettersen, orthopaedic resident, SIHT, Gjøvik.  Ove Talsnes, consultant orthopaedic surgeon, SIHT, Elverum  Jonas Larsen Hilmo, Nordlandssykehuset Bodø |
|  | 3b | Name and contact information for the trial sponsor:  South-Eastern Norway Regional Health Authority  E-mail: [Forskningsmidler@helse-sorost.no](mailto:Forskningsmidler@helse-sorost.no) Phone +47 62 58 55 00 |
|  | 3c | Role of trial sponsor and funders in design, conduct, analysis, and reporting of trial; including any authority over these activities: The trial sponsor finances the project after application based on the trial protocol. The sponsor requires annual reports on number of patients included and major adverse events registered. Only Ethical Board-approved projects are considered for financing from the Norwegian Health Authorities. Østfold Hospital Trust holds annual follow-up meetings and consultants from its research department assists the formal work of writing and signing agreements between the attending hospitals and other institutions involved. The sponsor is not involved in the performance of the trial. NTNU is attending agreements for financing Open Acces publications between Norwegian governmental institutions and international publishing companies. |
|  | 3d | Composition, roles, and responsibilities of the coordinating site, steering committee, endpoint adjudication committee, data management team,  and other individuals or groups overseeing the trial:  Østfold Hospital Trust carries the main responsibility for organizing and coordinating the research project. The hospital trust’s research department organizes the project which also is well integrated in the department of orthopaedic surgery. Norwegian University of Science and Technology (NTNU). NTNU has a data transfer agreement with Hemit, an information technology corporation that runs the research data base eFORSK. Hemit is owned by Central Norway Regional Health Authority. NTNU and ØHT are main institutions for the trial and have agreed upon shared responsibility for its data management. The National Health Network “Norsk Helsenett” is used by all attending hospitals in Norway and allows access to the common database in eFORSK. The PhD candidate has the responsibility for the day-to-day management of the project, supported by the main supervisor and the project leader. The project group routinely reports to the Data Safety and Monitoring Board. |
| **Open science** | | |
| Trial registration | 4 | ClinicalTrials.gov # 2024104, registered the 29^th^ of April 2024. |
| Protocol and statistical  analysis plan | 5 | ClinicalTrials.gov # 2024104 |
| Data sharing | 6 | Participant files will be maintained in storage for a period of five years after the completion of the study before deletion. Deidentified data will be stored in the NICE-1 network of the NTNU and shared on reasonable request. |
| Funding and conflicts of interest | 7a | South-Eastern Norway Regional Health Authority has granted ØHT 50% employment of the PhD candidate for 6 years from August 2024. The research department of ØHT has guaranteed to cover costs for trial-related x-ray surveys, open access publishing and presenting our research at international conferences. |
|  | 7b | The project leader has received lecturing fees from Amgen and UCB. Co-author Skorpa Nilsen has received lecturing fees from Smith & Nephew. |
| Dissemination policy | 8 | Results will be communicated to attending patients, presented in regional media and published in international peer-reviewed journals and presented at national and international conferences. Both positive and negative results will be reported, and co-authors will meet the criteria for co-authorship as defined by the International Committee of Medical Journal Editors. Results from five years follow-up will be published separately. |
| **Introduction** | | |
| Background and  rationale | 9a | A major number of ankle fractures treated in former study samples were less severe and would be treated conservatively with current guidelines. This supports the need for studies on fractures still chosen for surgery with current guidelines, a group of more severe fractures than in the samples most research has been done on until now.  The trial presented is the first major RCT on deep posterior deltoid ligament repair in Weber B SER4B ankle fractures. Our trial may give crucial knowledge on whether additional deltoid ligament repair preserves function better than the established treatment with lateral fracture repair and sometimes a trans-syndesmotic fixation**.**  Smaller studies have documented that medial ligament repair is a good option for regaining ankle joint anatomy. This repair also compensates for syndesmotic injury^[13](https://sciwheel.com/work/citation?ids=8486088&pre=&suf=&sa=0&dbf=0)^. Pakarinen et al showed that lateral malleolar fractures Weber B SER4 with a positive external rotation test after bony fixation did not benefit from a trans-syndesmotic screw.  Deltoid ligament repair has been shown to give a more predictable reduction of the tibiofibular syndesmosis than performing a direct trans-syndesmotic fixation and considerably less frequent reoperations for hardware removal. The distal tibiofibular syndesmosis and deltoid ligament have a synergistic effect in ankle stability. When to fix, which or both, is not clear, but fixing one of them seems to reduce the need of fixing the other. As far as we know, no gold standard exists for operative treatment of SER4B fractures. |
|  | 9b | Explanation for choice of comparator: We have chosen lateral plating only as comparator and additional deltoid ligament repair as intervention.  Different options have been discussed before choosing treatment arms, also simply deltoid ligament repair without plating of the lateral malleolus as an intervention, to turn a Lauge-Hansen SER4B into a SER2 fracture, a fracture that could be treated without surgery. This suggestion was found to be too controversial.  We know that a trans-syndesmotic fixation has a synergistic effect to deltoid ligament repair in stabilizing the ankle fork around the talus, and probably holding the torn deltoid ligament ends in proximity in most cases. Several of us have seen an ankle still being unstable after lateral plating, where the pathological medial talar tilt sometimes diminishes after a trans-syndesmotic fixation.  We see the risk of trans-syndesmotic fixations hiding the potential effect of deep posterior deltoid ligament repair.  Nor do we know the effect of trans-syndesmotic fixation in a sample of SER4B fractures. This could have been a third arm of treatment. At the same time, we know that trans-syndesmotic fixations carry a high malreduction rate and the need for implant removal is quite frequent. When we were about to start the trial, realising that established practice differs in the attending hospitals, we could not defend prohibiting the use of trans-syndesmotic implants in either group. The chosen arms of treatment attempts to be a pragmatic approach where the main difference between the groups will be whether the deep posterior deltoid ligament is repaired or not. We acknowledge that the solely plating treatment arm may contain several trans-syndesmotic fixations that may compensate for and conceal the true benefit from deltoid ligament repair compared to plating only. |
| Objectives | 10 | Some side effects from deltoid ligament repair as an additional step in a surgical procedure may be expected. Side effects can be pain from the posterior tibial tendon or ankle stiffness or problems from the ankle or subtalar joint after deltoid ligament suture in case of malplacement of the ligament suture anchor. Although not yet an everyday procedure, deltoid ligament repair might be challenging for anyone other than foot and ankle surgeons or experienced trauma surgeons. The protocol states that one of such surgeons are to take part in the surgical procedures. We do not find deltoid ligament repair difficult to teach. We believe the moderate risk implied by this ligament suture may be outweighed by improved talar reduction, ankle stability and function. All surgeons picked to perform the procedures in collaborating hospitals have been offered extra teaching and cadaveric deltoid ligament repair procedure training. |
| **Methods: Patient and public involvement, trial design** | | |
| Patient and public  involvement | 11 | Patients have been interviewed on the content in the PROMs and hence contribute to the development of Norwegian translations. A user representative from the Consumer board of ØHT has given constructive feedback on layout and content on written patient information |
| Trial design | 12 | This is a preregistered ongoing multicentre randomized controlled trial (computerized) with clinical superiority design. We aim to show whether deltoid ligament suture gives a superior result to solely osteosynthesis of the lateral malleolus in unstable Weber B/Lauge-Hansen Supination External Rotation 4b (SER4b) ankle fractures. |
| **Methods: Participants, interventions, and outcomes** | | |
| Trial setting | 13 | Patients will be included from the area of Sykehuset Levanger, Nordlandssykehuset Bodø, Sykehuset Innlandet Gjøvik & Elverum, Haukeland University Hospital, Ålesund Hospital, Stavanger University Hospital, Oslo University Hospital and Sykehuset Østfold. These are both local hospitals, regional and national trauma hospitals. The catchment areas of the participating centres comprise more than 2,000,000 persons. All four main regions of the Health Authorities in Norway are represented. |
| Eligibility Criteria | 14 | \| Patients are eligible if they present to one of the participating hospitals and comply with the inclusion and exclusion criteria. \| \| \| --- \| --- \| \| **Inclusion criteria** 18-65 years of age at presentation \| **Exclusion criteria** \| \| Initial medial clear space (MCS) ≥7mm or weightbearing x-ray evaluated as unstable (side to side difference >1 mm ***or*** \| Assumed not compliant (drug use, cognitive- and/or psychiatric disorders). \| \| Fracture dislocation (*when doubt about state prior to reduction shall WBXR be performed*) \| Insufficient language skills (Scandinavian) \| \| Able to walk without aids before the injury \| Multi-trauma or pathologic fracture \| \| Posterior malleolus fragment Mason & Molloy 1 or no posterior malleolus fragment^[15](https://sciwheel.com/work/citation?ids=16610910&pre=&suf=&sa=0&dbf=0)^ \| Neuropathies and symptomatic generalized joint disease such as Rheumatoid Arthritis \| \| Surgery planned within 2 weeks after injury and available for follow up \| Previous ipsilateral former ankle surgery or fracture or previous injury with marked sequela of the lower limb \| \| No syndesmotic screw or suture button planned prior to the surgical procedure \| Open fx Gustilo-Anderson II or more^[23](https://sciwheel.com/work/citation?ids=3193922&pre=&suf=&sa=0&dbf=0)^ or other medial soft tissue problem considerably increasing risk of additional medial approach to the ankle. \| \| No other more severe condition in the same extremity \| |
| Intervention and comparator | 15a | Surgical technique common for both treatment arms:  The lateral malleolus shall be fixed with a plate respecting the principles of modern fracture treatment. Particularly in cases with several fragments or expecting inferior bone density, we recommend modern plates with angular stability. For the intervention group deltoid ligament repair should be performed as described in the protocol appendix. |
|  | 15b | After lateral plating further testing is optional, i.e. talar shift and tilt or other tests to reveal syndesmotic instability. |
|  | 15c | As far as the intervension is the one particular surgical operation, no particular strategies are needed to adhere to the allocated arm of treatment. In case of treatment failures and need for reoperations because of medial or syndesmotic ankle instability, this will be addressed at the one-year follow-up or if needed on earlier supplementary outpatient contacts. |
|  | 15d | Preoperative CT should be performed as a general routine in cases of suspicion of additional injuries. Indication for further radiological surveys like MRI or postoperative CT is set by the treating surgeons. Concomitant care: If the surgeon notices severe instability that he or her finds compulsory to address further, like syndesmotic instability, this should be performed, and actions noted in the surgery protocol. |
| Outcomes | 16 | Primary outcome: Patient-reported function in OMAS score after lateral malleolar plating only versus additional deltoid ligament suture at 1 and 2 years. Results at 12 weeks will also be reported. Secondary outcomes:  -Infection, reoperation and other major adverse events shall be registered and compared between groups.  -Difference in medial clear space on WBXRs or gravity stress images after surgery with or without deltoid ligament repair. Sectra Picture Archiving and Communications System (Sectra AB, Linköping, Sweden) will be used for radiographic measurements.  -Signs of post-fracture arthritis is another important outcome and will be reported according to Kellgren Lawrence Scale.  -Function reported by Ankle Fracture Outcome of Rehabilitation Measure (A-FORM) Self-Reported Foot and Ankle Score (SEFAS) Patient-Reported Outcomes Measurement Information System (PROMIS), Visual Analogue Scale (VAS) pain and Euroquol EQ-5D 5L. The former is generic; referring to the individuals general health, OMAS and the other named Patient Reported Outcome Measures (PROMs) are specific to the anatomic region. |
| Harms | 17 | Harms are major complications to injury, surgery or anaesthesia like pulmonary embolus or myocardial infarction. Deep surgical infections, fixation failure or other treatment failures requiring reoperations. Complications will be discussed among local investigators and the steering group in search for avoidable causes and best possible follow up. Harms will be reported to the DSMB. Actions to prevent them in the topical institutions and other recruiting institutions will be made. If several occur recruitment may be stopped locally or in general. |
| Participant timeline | 18 | Refer to Figure 1 at the end of the document: SPIRIT 2025 diagram of the schedule of enrolment, interventions, and assessments |
| Sample size | 19 | To detect a defined minimal clinically important difference of between 8 and 10 (slightly more than half of the standard deviation (SD), or 9,7 as referred by McKeown et al (*Physiotherapy 2021*)) in Olerud-Molander ankle score between the study-groups, expected SD 14 points (Pakarinen (*FAI 2011*) et al found 13 and 15 in their groups, Molund et al had a SD of 12 on average (*Clin Rehabil. 2020*), significance level *α* = 0*.*05, Power 0,8, and estimated 20% drop out (two-sided t-test, clinical superiority design) we will include 60 patients in each group to ensure sufficient power after expected drop out (Zhong et al, *J Thorac Dis. 2009*)*.* |
| Recruitment strategy | 20 | Statistics from our surgical departments suggest that more than 100 candidates are eligible for inclusion in our recruitment districts a year. Patients arriving at recruiting hospitals receive oral and written information about the trial from the orthopaedic resident surgeon on call or a physiotherapist or nurse in the emergency department or outpatient clinic. It will be stressed that participation is voluntary, and that they can withdraw their consent at any time without influencing their further treatment. |
| **Methods: Assignment of interventions** | | |
| Randomisation: | | |
| Sequence generation | 21a | Local investigators collect the computerized randomization sequence. If not available, it will be done by the steering committee and message brought by telephone. |
|  | 21b | A total of 120 patients will be randomized in a 1:1 ratio. Stratification for block randomisation will be by biological sex and age above or below 50 years at the time of inclusion. |
| Allocation concealment mechanism | 22 | Computerized held in the eFORSK database (Hemit), Norsk Helsenett. |
| Implementation | 23 | Local investigators and other colleagues directly involved in the treatment of included patients will be informed about their allocation |
| Blinding | 24 | Not applicable. Sham surgery was not considered for the comparator group. As suture anchors used mostly contain metal that will be visible on x-rays, blinding for radiological measurements will not either be possible. |
| **Methods: Data collection, management, and analysis** | | |
| Data collection methods | 25a | Information from the operation protocols and patient records from the hospital stay will be used to collect descriptive data for comparison between allocation groups. All data will be entered and stored electronically in the electronic research database (eFORSK, Hemit, Trondheim, Norway), both from outpatient clinic follow-up visits and x-ray measurements. The research database also collects PROM-data electronically from the patients at follow-up beyond outpatient clinic visits. Data will be deidentified before export for statistical analysis. |
|  | 25b | Information from the operation protocols and patient records from the hospital stay will be used to collect descriptive data for comparison between allocation groups. All data will be entered and stored electronically in the electronic research database (eFORSK, Hemit, Trondheim, Norway), both from outpatient clinic follow-up visits and x-ray measurements. The research database also collects PROM-data electronically from the patients at follow-up beyond outpatient clinic visits. Data will be deidentified before export for statistical analysis. |
| Data management | 26 | The dedicated lead investigators at collaborating hospitals try to perform most of the outpatient follow-ups of their patients. The PhD candidate surveils controls at all attending hospitals and has contact data to check if follow-up questionnaires are received and remind patients to respond to these. Link to the data management plan: <https://dmp.sikt.no/plan/8678ef77-fad1-4bdf-8a74-3c07341ede2c> |
| Statistical methods | 27a | Access to the study data will be restricted to study group members, where the hospitals lead investigators reach their own hospitals’ data through the national health network by two-factor authentication. Data will be deidentified before export for statistical analysis. |
|  | 27b | Descriptive data will be presented as means with standard deviations, medians with range or frequencies, and percentages when appropriate. T-test will be used to analyse differences between groups for the primary and secondary outcomes. A non-parametric test will be used if data are skewed. Data will be analysed using the IBM SPSS Statistics and STATA (StataCorp). |
|  | 27c | All randomized participants will be included in the analyses as far as topical information is collected. |
|  | 27d | Patients with missing data will not be included in the analyses for where certain data is missing. Electronical reporting systems does not allow incomplete answers. We have estimated the need of up to 50 patients in each group for possible significant answers. Including 60 in each of the two groups permits a loss to follow-up of 20%. A link to the statistical analysis plan can be found at: <https://www.sykehuset-ostfold.no/kliniske-studier/skal-vi-reparere-deltoidligamentet-ved-ustabile-ankelbrudd-funksjon-rapportert-av-pasienter-etter-operativ-behandling.-stabilitetsmal-pa-rontgenbilder> |
| Data monitoring  Committee | 28a | Comparisons will also be done between different groups of stratification (sex and age above or below 50 at the time of presentation) and the 25% of patients having the worst outcome in each treatment arm. The study is underpowered to expect significant findings from the subgroup analyses. All statistical analyses will be performed in cooperation with the statistician at ØHT and NTNU. |
|  | 28b | Are H. Stødle, MD, PhD, consultant orthopaedic surgeon, Oslo University Hospital and Mette Andersen, MD, PhD, consultant orthopaedic surgeon, Aleris Hospital, Tromsø and Vestre Viken HT, have a mandate to stop the study if untoward effects are observed. |
| Trial monitoring | 29 | Major adverse events like peroperative complications, reoperations, infection or other complications or side effects are observed during primary hospital stay, 6- or 12-weeks outpatient control or later. Adverse events will be reported every 6 months, and preliminary results from the first 30 patients 12 weeks follow-up presented. Interim analysis of patient-reported functional outcome and adverse events each treatment arm after 60 patients at follow-up of 1 year. |
| **Ethics** | | |
| Research ethics  Approval | 30 | The Regional Committee for Medical and Health Research Ethics (REK Sørøst, ref. 496556) and the Data Protection Officers at all collaborating hospitals have approved patient recruitment to this trial. |
| Protocol amendments | 31 | Protocol modifications near the very beginning of inclusion was done by phone contact, written recruitment procedure and surgical procedure descriptions by e-mail. Lectures were given about the trial to all attending hospitals by the local coordinators or the PhD candidate. |
| Consent or assent | 32 | Patients arriving at recruiting hospitals receive oral and written information about the trial from the orthopaedic resident surgeon on call or a physiotherapist or nurse in the emergency department or outpatient clinic. Participants will be informed, both written and orally, that participation in the studies is voluntary, and that they can withdraw their consent at any time without influencing their further treatment. |
| Confidentiality | 33 | Access to the study data will be restricted to study group members, where the hospitals lead investigators reach their own hospitals’ data through the national health network by two-factor authentication. Data will be deidentified before export for statistical analysis and publishing. |
| Ancilliary and post-trial care | 34 | Patients will be followed up as usual after treatment in our departments. If untowards effects and delayed recovery after unexpected side effects or complications, they may apply for economic compensation after loss of labour capacity at npe.no |
| SPIRIT=Standard Protocol Items: Recommendations for Interventional Trials. | | |

| **Figure 2 \| SPIRIT 2025 diagram of the schedule of enrolment, interventions, and assessments** | | | | | |
| --- | --- | --- | --- | --- | --- |
| **Time point** |  | | | | |
| **Enrolment** | Screening for eligibility | | See Eligibility criteria (Table 1). | | |
|  | Informed consent | | Written and oral information from a physiotherapist, nurse or orthopaedic surgeon other than the operating. | | |
|  | Randomization | | Randomization module in the eFORSK database (Block randomization) | | |
|  | | | | | |
| **Surgical intervention**  (within 2 weeks after injury) | Intervention:  *Additional deltoid ligament repair* | | Surgical treatment will be performed by equally experienced surgeons in both the intervention and comparator groups: Fellowship-trained foot and ankle or trauma surgeons with lower extremity experience.  Observations from hospital stay and operation: Injury and repair description, duration of surgery. Per- and perioperative complications, length of hospital admission. | | |
|  | Comparator:  *Plating of the lateral malleolus only* | |  |  |  |
|  | | | | | |
| **Rehabilitation protocol** | Equal for both study arms | | Up to two weeks in cast or Walker boot and partial weight-bearing 20 kg. Thereafter movement exercises and weightbearing as tolerated. Orthosis optional from two weeks. | | |
|  | | | | | |
| **Assessments** | | | | | |
| **Time of follow-up** | **WBXR*** | **Gravity stress XR** | | **PROMs** (e-survey) | **Other** |
| 6 weeks ± 10 days | X |  | |  | VAS pain, adverse events. Use of orthosis and supporting aids. |
| 12 weeks ± 2 w | X |  | | X | As for 6w. Time of return to work. |
| 1 year ± 1 month | X | X | | X** | Validation of test-retest properties. |
| 2 years ± 2 m | X |  | | X | Last observation for main publication |
| 5 years ± 5 m | X | X | | X | Will be published in a later paper |

*Weight-bearing x-rays (WBXR) of both legs in the mortise projection and measurement of MCS at all times of follow-up.
**Sent twice for test-retest validation of the PROMs used in Norwegian versions, in particular the Ankle Fracture Outcome of Rehabilitation Measure (A-FORM) translation.
VAS pain =Visual Analogue Scale pain
Gravity stress x-rays (after Michelson, *Clin Orthop Relat Res.* 2001 and Schock et al, *JBJS(Br)* 2007) performed at 1- and 5-years follow-up for evaluation of superficial deltoid ligament integrity at one and five years). Adverse events as peroperative complications, thrombosis, surgical site infection, other postoperative complications and reoperations are registered. Posttraumatic arthritis is registered according to the Kellgren and Lawrence scale.
