# Supplementary material for: The Benefit of Repairing the Deltoid Ligament in Unstable Ankle Fractures: Patient-Reported Functional Outcome and Radiological Stability Measurements; a Clinical Trial Protocol
Source: Foot Ankle Orthop. 2025 Nov 12;10(4):24730114251386735. doi: 10.1177/24730114251386735 (PMC12615956; doi:10.1177/24730114251386735)
Supplement: sj-docx-5-fao-10.1177_24730114251386735 – Supplemental material for The Benefit of Repairing the Deltoid Ligament in Unstable Ankle Fractures: Patient-Reported Functional Outcome and Radiological Stability Measurements; a Clinical Trial Protocol [file sj-docx-5-fao-10.1177_24730114251386735.docx]

***“The benefit of repairing the deltoid ligament in unstable ankle fractures:****Patient-reported functional outcome and radiological stability measurements.”*

NCT# 06568276

Approved by the Regional Committee for Medical and Health Research Ethics (REK Sør-øst) 23^rd^ of January 2025 after extended number of including hospitals.

**Project Title:**

***The benefit of repairing the deltoid ligament in unstable ankle fractures:****Patient-reported functional outcome and radiological stability measurements.*

**Abstract**

**Aims**

Suturing the deep posterior deltoid ligament in unstable ankle fractures is novel to established treatment. There is reason to believe that deltoid ligament repair in addition to plating of the lateral fracture will improve stability restoration. Cadaveric studies on Weber B fracture complex models have shown promising results. We will perform a randomized controlled multicentre trial to show whether suturing the deep deltoid ligament can contribute to better functional results. Whether the ligament repair affects radiological stability parameters and the incidence of ankle joint osteoarthritis on long term, will also be investigated.

**Methods**

A total of 120 patients with Lauge Hansen SER 4B ankle fractures will be randomized (1:1 ratio) to conventional plating of the lateral malleolus only or additional suture of the deep deltoid ligament. Main end point will be function measured in Olerud-Molander score (OMAS) at 1 and 2 years. Secondary end points are Self-Reported Foot and Ankle Score (SEFAS), Ankle Fracture Outcome of Rehabilitation Measure (A-FORM), VAS pain and Euroquol EQ 5D-5L. Differences on group level in medial clear space (MCS), side to side differences from weightbearing ankle x-rays (WBXR) and gravity stress X-ray, rates of treatment-related adverse events and incidence of posttraumatic arthritis will be reported.

**Discussion**

Several patients sustaining severe ankle fractures have shown a considerable loss of function including ADL function compared to less severe fractures. Improving treatment outcome may also be beneficial by preserving working capacity and quality of life and thereby reducing community expenses. The objective of this RCT is the contribution from repair of the deep deltoid ligament in unstable Weber B ankle fractures. Because of increasing attention to deltoid ligament patency as fundamental to ankle joint stability particularly in a fracture setting, lately increasing interest has been paid to repairing the medial ankle ligament complex. This both as an option or an add to osteosynthesis of the lateral malleolus and trans-syndesmotic fixation.

**Registration details**
ClinicalTrials.gov # 2024104.

**Introduction**

Ankle fractures occur in 1 out of up to 800 persons a year and are among the most frequent orthopaedic injuries.[^27^](https://sciwheel.com/work/citation?ids=16605604&pre=&suf=&sa=0&dbf=0) Post-traumatic arthritis occurs after fractures and sprains and causes persistent stiffness and pain in affected joints. The risk of developing posttraumatic arthritis is linked to the severity of the fracture and joint stability after treatment.[^41^](https://sciwheel.com/work/citation?ids=6894833&pre=&suf=&sa=0&dbf=0)[^39^](https://sciwheel.com/work/citation?ids=17402262&pre=&suf=&sa=0&dbf=0) Arthroplasty in the ankle is by now less successful compared to hip and knee.[^1^](https://sciwheel.com/work/citation?ids=17379713&pre=&suf=&sa=0&dbf=0) Prevention by optimal trauma care will be preferable to treatment of established arthritis.

During the last few decades less severe ankle fractures have been shown not to need operative treatment in general.[^10,16^](https://sciwheel.com/work/citation?ids=16605923,16605570&pre=&pre=&suf=&suf=&sa=0,0&dbf=0&dbf=0) The total number of ankle fracture surgeries has decreased. However, how to treat the more complex fractures is still a challenge. Surgically treated fractures nowadays are on average more complex than in samples from the three recent decades. The use of weightbearing x-rays (WBXR) in stability assessment of ankle fractures is now established. This is by now well documented as a main guide in the choice between conservative and surgical treatment.[^14,38,43^](https://sciwheel.com/work/citation?ids=16605935,8493016,8486400&pre=&pre=&pre=&suf=&suf=&suf=&sa=0,0,0&dbf=0&dbf=0&dbf=0) The understanding of these injuries implies a recognition of the role of the deep deltoid ligament as a main stabilizer of the ankle joint.

Cadaveric studies have shown that, as a single procedure, repair of the deep posterior deltoid ligament tends to give more stability than the osteosynthesis of the lateral malleolus. Combining the ligament suture and the lateral plating increases stability to a large extent.[^9,15^](https://sciwheel.com/work/citation?ids=16606455,17016014&pre=&pre=&suf=&suf=&sa=0,0&dbf=0&dbf=0)[^28^](https://sciwheel.com/work/citation?ids=11669716&pre=&suf=&sa=0&dbf=0)[^3^](https://sciwheel.com/work/citation?ids=16605925&pre=&suf=&sa=0&dbf=0)

Repairing the deltoid ligament is an adjunct to already established practice. We see a potential in this additional ligament repair. Clinical support already exists that it seems to have a role in treatment of unstable ankle fractures on group level.[^36,39,42^](https://sciwheel.com/work/citation?ids=17402262,15757907,16966498&pre=&pre=&pre=&suf=&suf=&suf=&sa=0,0,0&dbf=0&dbf=0&dbf=0) The authors have experienced individual cases where deltoid ligament repair has been necessary because of evident medial ankle instability after lateral fracture fixation.

*Anatomy of the deltoid ligament and its biomechanical properties*

In ankle fractures, partial tears of the deltoid ligament are not rare. Whether there is *a complete deltoid ligament injury* or not helps to separate fractures requiring surgery from the rest. The literature on the deltoid ligament reveals inconsistency in description and interpretation of its anatomy and biomechanics.[^4,11,13,19^](https://sciwheel.com/work/citation?ids=3071351,5800833,16605648,508039&pre=&pre=&pre=&pre=&suf=&suf=&suf=&suf=&sa=0,0,0,0&dbf=0&dbf=0&dbf=0&dbf=0) Several of the patient samples in clinical reports are a mix of partial and complete ligament injuries. The way of repairing differs,[^23,24,33,39,46^](https://sciwheel.com/work/citation?ids=17778579,16966208,17778589,17402262,16605927&pre=&pre=&pre=&pre=&pre=&suf=&suf=&suf=&suf=&suf=&sa=0,0,0,0,0&dbf=0&dbf=0&dbf=0&dbf=0&dbf=0)and also has not been clearly described in some studies.[^18,40^](https://sciwheel.com/work/citation?ids=16605624,17779599&pre=&pre=&suf=&suf=&sa=0,0&dbf=0&dbf=0)


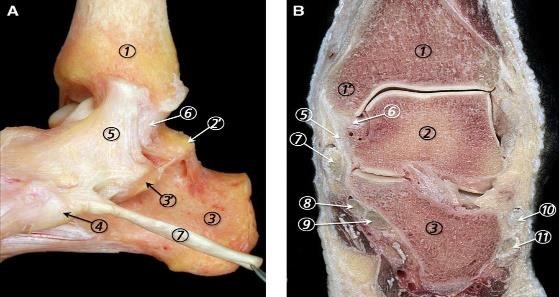
**Figure 1** (*A*) Medial view of the ankle joint ligaments showing their typical fanlike morphology. (*B*) Frontal section of the ankle joint where the superficial and deep layers of the MCL (medial collateral ligament) are separated by a small mass of fatty tissue. 1, tibia; 1’, medial malleolus; 2, talus; 2’, medial talar process; 3, calcaneus; 3’, sustentaculum tali; 4, navicular tuberosity; 5, superficial layer of the MCL; 6, deep layer of the MCL; 7, tibialis posterior tendon; 8, flexor digitorum longus tendon; 9, flexor hallucis longus tendon; 10, peroneus brevis tendon; 11, peroneus longus tendon. (Golano et al[^13^](https://sciwheel.com/work/citation?ids=3071351&pre=&suf=&sa=0&dbf=0))

Standard operative treatment of Weber B ankle fractures has been plating of the distal fibula, screw fixation of the medial malleolus and, to a varying extent, syndesmotic fixation. Distal fibular fracture in combination with a full deltoid ligament tear has been termed «bimalleolar equivalent ankle fracture». It may seem logical and tempting to fix medial malleolar fractures that are evident on x-rays, and this practice has been less disputed. During surgery, when residual instability is found in ankle fractures after fixation of the malleoli, the established strategy has been restabilizing the distal tibiofibular joint/syndesmosis.[^28^](https://sciwheel.com/work/citation?ids=11669716&pre=&suf=&sa=0&dbf=0) This has in general been done by a trans-syndesmotic fixation using a screw or suture button and/or fixation of avulsed syndesmosis-bearing fragments. Frankly, the rationale of not fixing the deltoid ligament is based on the presumption that this is not necessary, unless the ligament is interposed in the medial gutter and obstructing reduction of the ankle joint.

Deltoid ligament repair is documented to be a good option to regain ankle joint anatomy from smaller studies. This repair also compensates for syndesmotic injury.[^21^](https://sciwheel.com/work/citation?ids=8486088&pre=&suf=&sa=0&dbf=0) Pakarinen et al showed that lateral malleolar fractures Weber B SER4 with a positive external rotation test after bony fixation did not profit on a trans-syndesmotic screw.[^32^](https://sciwheel.com/work/citation?ids=5238703&pre=&suf=&sa=0&dbf=0)

The effect of deep deltoid ligament repair in Weber B ankle fractures and its effect on long term function and arthritis is not yet known from larger clinical studies. Deltoid ligament repair has also been shown to give a more predictable reposition of the tibiofibular syndesmosis than performing a direct trans-syndesmotic fixation and considerably less frequent reoperations for hardware removal.[^39,44,45^](https://sciwheel.com/work/citation?ids=16605596,17402262,17402252&pre=&pre=&pre=&suf=&suf=&suf=&sa=0,0,0&dbf=0&dbf=0&dbf=0) The distal tibiofibular syndesmosis and deltoid ligament have a synergistic effect in ankle stability.[^24^](https://sciwheel.com/work/citation?ids=16605927&pre=&suf=&sa=0&dbf=0) When to fix, which or both, is not clear, but fixing one of them seem to reduce the need of fixing the other. As far as we know, there is no defined gold standard for operative treatment of SER4B fractures.

**Clinical Relevance**

- A major number of fractures treated in former study samples were less severe and would be treated conservatively with current guidelines. This supports the need for studies on fractures still chosen for surgery with current guidelines, a group of more severe fractures than in the samples most research has been done on until now.
- The trial presented is the first major RCT on deep posterior deltoid ligament repair in Weber B SER4B ankle fractures. Our trial may give crucial knowledge on whether additional deltoid ligament repair preserves function better than the established treatment with lateral fracture repair and sometimes a trans-syndesmotic fixation**.** Whether medial ankle joint congruency is reestablished and if deltoid ligament patency can be restored, will also be investigated. And can this help us avoid post-fracture arthritis?

**Study objectives**


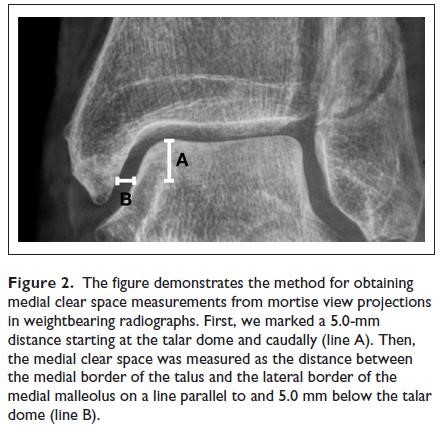
We expect that additional deltoid ligament suture is superior to solely plating of the lateral malleolus when taking minimal important difference (MID) into consideration.

Primary hypothesis: «Patient-reported function in Olerud Molander Ankle Score (OMAS) [^31,34^](https://sciwheel.com/work/citation?ids=11398418,17204320&pre=&pre=&suf=&suf=&sa=0,0&dbf=0&dbf=0) will be clinically superior to after only plating of lateral malleolar fractures in Lauge Hansen SER 4b fractures one and two years after surgery»

The primary hypothesis will also be tested for patient-reported function in A-FORM and SEFAS as they give more nuanced responses and have a less categorical scoring system than the OMAS.

Secondary hypotheses: “The difference in medial clear space measurements of injured compared to non-injured side on weightbearing x-rays is significantly different between groups” and

“The difference between groups in medial clear space change measured in mm from weightbearing x-rays to gravity stress images of injured side is statistically significant at one or two years”.

A significant difference of medial clear space is set to be >1,0 mm. (Figure 2[^16^](https://sciwheel.com/work/citation?ids=16605570&pre=&suf=&sa=0&dbf=0) and 4[^37^](https://sciwheel.com/work/citation?ids=13145447&pre=&suf=&sa=0&dbf=0))

**Methods**

**Study design**This is the research protocol for a preregistered ongoing multicentre randomized controlled trial (computerized) with clinical superiority design. We aim to show whether deltoid ligament suture gives a superior result to solely osteosynthesis of the lateral malleolus in unstable Weber B/Lauge-Hansen Supination External Rotation 4b (SER4b) ankle fractures. The protocol is developed in adherence with the Standard Protocol Items: Recommendations for Interventional Trials (SPIRIT) statement[^5,6^](https://sciwheel.com/work/citation?ids=18154596,594495&pre=&pre=&suf=&suf=&sa=0,0&dbf=0&dbf=0). When submitting this protocol, x patients were enrolled in the study. Inclusion started in September 2024, and we anticipate reaching our recruitment target of 120 patients within 2026.

**Study setting**Patients will be included by written consent after written and oral information. Patients will be included from the area of Sykehuset Levanger, Nordlandssykehuset Bodø, Sykehuset Innlandet, Haukeland University Hospital, Ålesund Hospital, Stavanger University Hospital, Oslo University Hospital and Sykehuset Østfold.

**Table 1. Eligibility criteria.**

**Interventions**

Surgery should be performed within 2 weeks after injury. Treatment in both arms of randomisation must be performed by equally experienced surgeons, a consultant or fellowship-trained orthopaedic surgeon taking part in the procedure, preferably a foot and ankle surgeon or experienced trauma surgeon. Preoperative CT should be performed as a general routine in suspicion of additional injuries.

**Surgical technique**

Both treatment arms: The lateral malleolus shall be fixed with a plate respecting principles of modern fracture treatment. Particularly in cases with several fragments or expecting inferior bone density, we recommend modern plates with angular stability. After lateral plating further testing is optional, i.e. talar shift and tilt or syndesmotic instability.

Concomitant care: If the surgeon notices severe instability that he or her finds compulsory to address further, this should be performed, and actions noted in the protocol.

Patients randomized to additional deltoid ligament repair: *Deltoid ligament repair is done by a curved incision following the path of the posterior tibial tendon from just proximal to the posterior limit of the medial malleolus till past the anterior tip. The tendon retinaculum is incised in the direction of the tendon. The tendon is lifted out of its sheath and held proximally. Note the degree of injury to the deep posterior deltoid ligament and site of tear (talar or tibial end). Because the ligament most often is torn on its talar end,*[^20^](https://sciwheel.com/work/citation?ids=6547876&pre=&suf=&sa=0&dbf=0) *the routine repair will be suturing the deep deltoid ligament fibres to a bone anchor in the talus. We recommend temporary pin placement and fluoroscopy in at least one projection (AP) to confirm correct positioning prior to anchor placement. A modern bone anchor must be used. The ligament should be tied sufficiently tight, if not neutral, the ankle is held in slight inversion and 10-15 degrees of plantar flexion when tightening and tying the sutures. Another suture anchor in the anterior colliculus of the medial malleolus is optional. We close more superficial and anterior parts of the ligament tear before putting the tendon back into its retinaculum, which is closed with a resorbable #1 suture or stronger.*

*We suggest medial ligament repair prior to lateral plating to get a better overview.*

Aftertreatment: Up to 2 weeks in a cast or walker orthosis when loading max 20 kg. Thereafter free movement with or without orthosis from 2 weeks, weightbearing as tolerated.

**Figure 3**


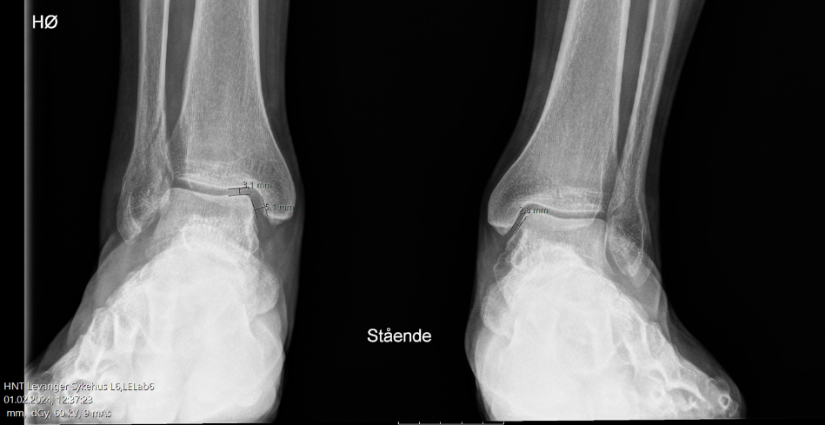

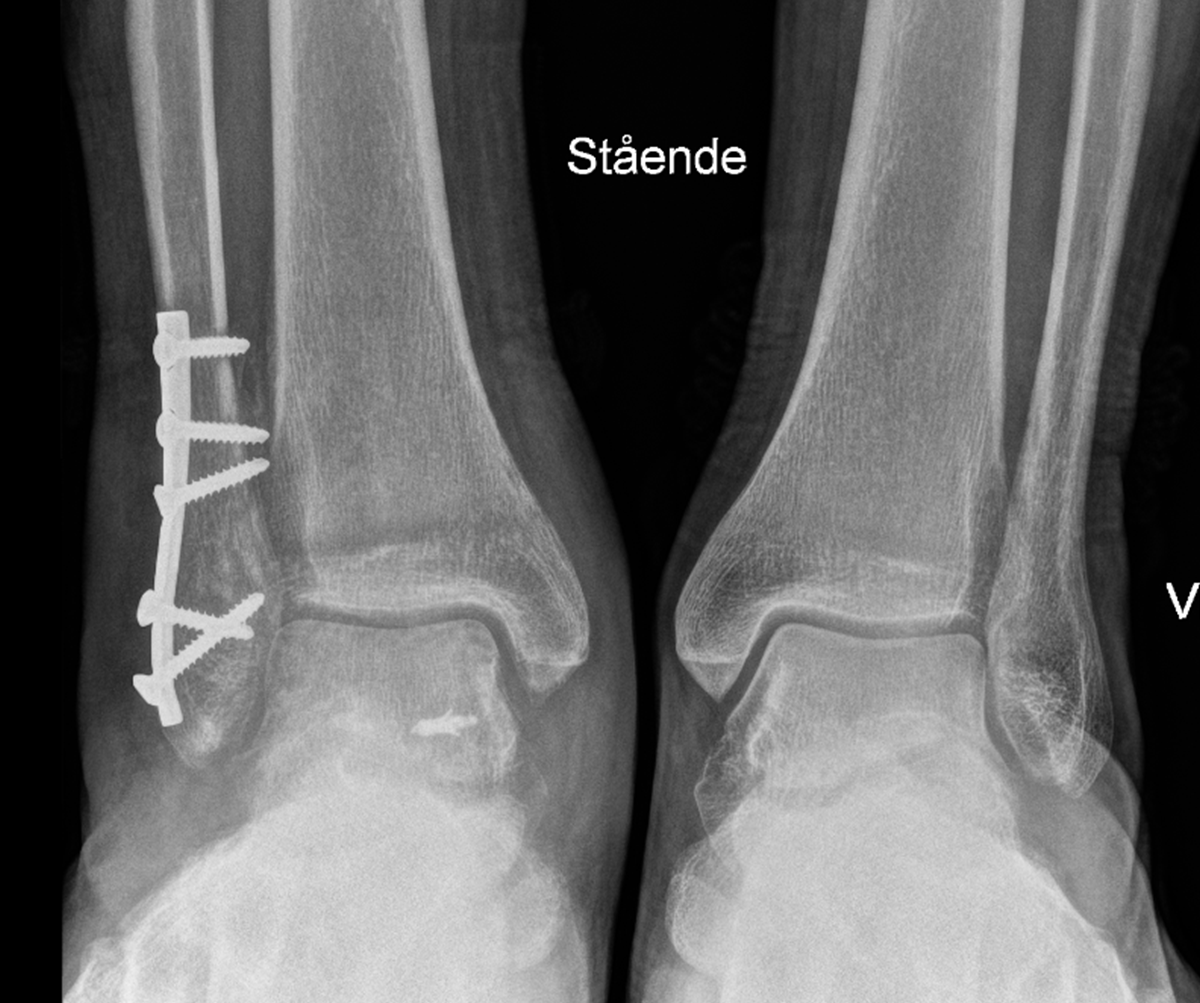

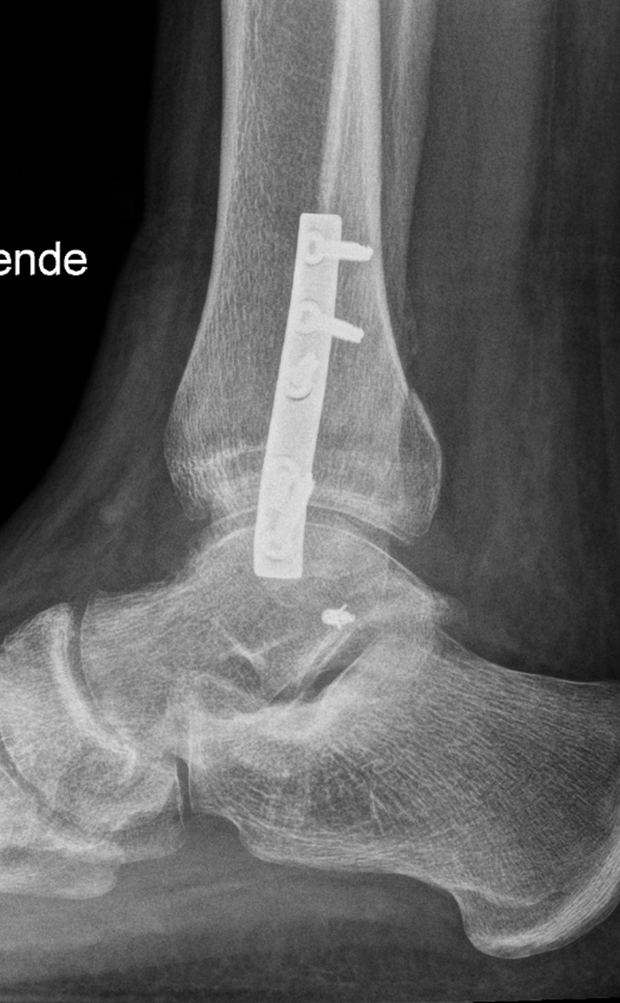


Picture showing weightbearing x-ray where MCS of the right ankle is about 2 mm increased compared to the non-injured left ankle. Deep posterior deltoid ligament sutured to the talus by an anchor placed just plantar to the posterior colliculus. Note the short distance from its attachment to the subtalar joint.

**Table 2. Patient timeline.**

| **Time of follow up** | **WBXR** | **Gravity stress** | **PROM (e-survey)** | **Other** |
| --- | --- | --- | --- | --- |
| **6 weeks** | X |  |  | VASpain, adverse events |
| **12 weeks** | X |  | X |  |
| **1 year** | X | X | X (sent twice*) | (*Validation study PROMs) |
| **2 years** | X |  | X |  |
| **5 years** | X | X | X |  |

WBXR of both legs in the mortise projection and measurement of MCS at all times of follow-up, and gravity stress x-rays (Figure 4) for evaluation of deltoid ligament integrity at one and five years)

**Figure 4** Gravity stress test after Schock et al[^37^](https://sciwheel.com/work/citation?ids=13145447&pre=&suf=&sa=0&dbf=0).


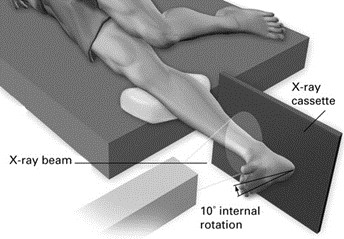


**Table 1 Eligibility criteria**

| Patients are eligible if they present to one of the participating hospitals and comply with the inclusion and exclusion criteria. | |
| --- | --- |
| **Inclusion criteria** 18-65 years of age at presentation | **Exclusion criteria** |
| Initial medial clear space (MCS)>=7mm or weightbearing x-ray evaluated as unstable (side to side difference >1 mm ***or*** | Assumed not compliant (drug use, cognitive- and/or psychiatric disorders). |
| Fracture dislocation (*when doubt about state prior to reposition shall WBXR be performed*) | Insufficient language skills (Norwegian) |
| Pre-injury walking ability without aids | Multi-trauma and pathologic fracture |
| Posterior malleolus fragment Mason & Molloy <2 or no posterior malleolus fragment[^25^](https://sciwheel.com/work/citation?ids=16610910&pre=&suf=&sa=0&dbf=0) | Neuropathies and symptomatic generalized joint disease such as Rheumatoid Arthritis |
| Available for surgery within 2 weeks after injury and available for follow up | Previous history of ipsilateral former ankle surgery or fracture or other injury with sequela of the lower limb |
| No syndesmotic screw or suture button planned prior to the surgical procedure, only on demand after peroperative assessment | Open fx Gustilo-Anderson II or more[^17^](https://sciwheel.com/work/citation?ids=3193922&pre=&suf=&sa=0&dbf=0) or other medial soft tissue problem considerably increasing risk of additional medial approach to the ankle. |
| No other more severe condition in the same extremity |  |

**Primary outcome**: Patient-reported function in OMAS score after lateral malleolar plating only versus additional deltoid ligament suture at 1 and 2 years. Results at 12 weeks will also be reported.
**Secondary outcomes**:
-Infection, reoperation and other major adverse events shall be registered and compared between groups.

-Difference in medial clear space on WBXRs or gravity stress images after surgery with or without deltoid ligament repair. Sectra Picture Archiving and Communications System (Sectra AB, Linköping, Sweden) will be used for radiographic measurements.

-Signs of post-fracture arthritis is another important outcome, and will be reported according to Kellgren Lawrence Scale[^22^](https://sciwheel.com/work/citation?ids=3806748&pre=&suf=&sa=0&dbf=0).

-Function reported by Ankle Fracture Outcome of Rehabilitation Measure (A-FORM) Self-Reported Foot and Ankle Score (SEFAS),[^7,8,12,30^](https://sciwheel.com/work/citation?ids=2909783,12136194,8509577,16606459&pre=&pre=&pre=&pre=&suf=&suf=&suf=&suf=&sa=0,0,0,0&dbf=0&dbf=0&dbf=0&dbf=0) Patient-Reported Outcomes Measurement Information System (PROMIS),[^2^](https://sciwheel.com/work/citation?ids=8052745&pre=&suf=&sa=0&dbf=0) Visual Analogue Scale (VAS) pain and Euroquol EQ-5D 5L.[^35^](https://sciwheel.com/work/citation?ids=1175040&pre=&suf=&sa=0&dbf=0) The former is generic; referring to the individuals general health, OMAS and the other named Patient Reported Outcome Measures (PROMs) are specific to the anatomic region. (Table 3)

**Table 3**

| **Type of PROM (scale)** | **Licence Y/N** | **#items (questions)** | **Foot (F) Ankle (A) or general (G)** | **Origin, comment** |
| --- | --- | --- | --- | --- |
| **OMAS** (0-100) | N | 9 | A | Most used |
| **SEFAS** (0-48) | N | 12 | FA | Swedish ankle registry |
| **A-FORM** (0-100) | Y (free) | 15 | A | Fracture specific |
| **EQ-5D 5L** (-0,59-1) | Y (free*) | 5+VAS health | G | General health |

Highest score is best, except from the A-FORM

The phenomenon of *ceiling effect* means high score in most patients answering a PROM. This has been a challenge in long term follow-up of in former studies on ankle fracture patients. When selecting PROMs as end points for our study, we have looked for PROMs asking for ability to fulfil difficult tasks to reach the highest score range. If the Norwegian version is ready at the end of 2025, we will also use the PROMIS Mobility bank 2.1 at one year follow up[^2^](https://sciwheel.com/work/citation?ids=8052745&pre=&suf=&sa=0&dbf=0).

**Sample size, power and stratification**

To detect a defined minimal clinically important difference of between 8 and 10 (slightly more than half of the standard deviation (SD), or 9,7[^26^](https://sciwheel.com/work/citation?ids=16606484&pre=&suf=&sa=0&dbf=0) as referred) in Olerud-Molander ankle score between the study-groups, expected SD 14 points (Pakarinen et al found 13 and 15 in their groups[^32^](https://sciwheel.com/work/citation?ids=5238703&pre=&suf=&sa=0&dbf=0), Molund et al had a SD of 12 on average[^29^](https://sciwheel.com/work/citation?ids=16606442&pre=&suf=&sa=0&dbf=0)), significance level *α* = 0*.*05, Power 0,8, and estimated 20% drop out (two-sided t-test, clinical superiority design) we will include 60 patients in each group to ensure sufficient power after expected drop out[^47^](https://sciwheel.com/work/citation?ids=2790916&pre=&suf=&sa=0&dbf=0). Stratification for block randomization will be by sex and age above or below 50 years at time of inclusion.

**Recruitment strategy and procedure**

Statistics from our surgical departments suggest that there will be more than 100 candidates a year prone for inclusion in our recruitment districts altogether. The catchment areas of the participating centres comprise more than 1,500,000 persons. Patients arriving at recruiting hospitals receive oral and written information about the trial from the orthopaedic resident surgeon on call or a physiotherapist or nurse in the emergency department or outpatient clinic. Participants will be informed, written and orally, that participation in the studies is voluntary, and that they can withdraw their consent at any time without influencing their further treatment.

**Concealment and blinding**

As no sham surgery will be performed to the medial side of the ankle in the osteosynthesis only treatment arm, and suture anchors often may be seen on x-rays, blinding will not be possible.

**Participants, organization and collaborations**

Project leader and co-supervisor: Y, MD, PhD, consultant orthopaedic surgeon, Østfold Hospital Trust (ØHT) and associate professor at Oslo University Hospital.

Main supervisor and principal investigator: YY, MD, PhD, consultant orthopaedic surgeon, Department of orthopaedic surgery, ØHT. YY has extensive clinical and research experience and is a reviewer of the journal Foot and Ankle International.

PhD candidate: X, consultant orthopaedic surgeon, Lead investigator at Sykehuset Levanger and PhD candidate in ØHT, will be responsible for the day-to-day management of the RCT.

Co-supervisors: Andrew M. Garratt, Dr. Scient, Norwegian Institute for Public Health (FHI). He has great experience in PROMs research and is the national main contact for EQ-5D and PROMIS.

Aksel Paulsen, MD, PhD, orthopaedic surgeon, head of research, associate professor, orthopaedic dept. Dr. Paulsen is lead investigator at Stavanger University Hospital and runs PROMs research.

XX, MD, PhD, consultant orthopaedic surgeon, Sykehuset Innlandet Hospital Trust (SIHT), Tynset and associate professor, Norwegian University of Science and Technology (NTNU). NTNU has a data transfer agreement with Hemit, an information technology corporation that runs the research data base eFORSK. Hemit is owned by Central Norway Regional Health Authority. NTNU and ØHT are main institutions for the trial and have agreed upon shared responsibility for its data management.

Lead investigators at other collaborating hospitals:

Z, consultant orthopaedic surgeon, Haukeland University Hospital

ZZ, consultant orthopaedic surgeon, Ålesund Sjukehus

ZZZ, consultant orthopaedic surgeon, Oslo University Hospital, Ullevål

ZZZZ, orthopaedic resident, ØHT

ZZZZ, orthopaedic resident, SIHT, Gjøvik.

ZZZZZ, consultant orthopaedic surgeon, SIHT, Elverum

ZZZZZZ, consultant orthopaedic surgeon, Nordlandssykehuset Bodø

Conflicts of interest: We claim no conflict of interest for the applicants.

**Data management, randomization and storage**

All data will be entered and stored electronically in the electronic research data base (eFORSK, Hemit, Trondheim, Norway). Access to the study data will be restricted to study group members, where the hospitals lead investigators reach their own data through the national health network by two-factor authentication. eFORSK holds a computerized randomization module. The research data base also communicates electronically to collect PROM-data from patients at follow-up. Data will be deidentified before export for statistical analysis. Participant files will be maintained in storage for a period of five years after the completion of the study before deletion.

**Implementation**

Lead investigators at recruiting hospitals randomize their patients to surgical treatment with or without deltoid ligament suture after consent before surgery in eFORSK and collect and plot information from treatment and first follow up at 6 weeks after surgery. Thereafter major adverse events and measurement from follow up x-rays are managed and reported locally at recruiting centres. The PhD candidate is responsible for conducting e-surveys, supporting the collaborating hospitals in recruitment of patients, treatment and follow up, and to conduct analysis and write manuscripts.

**Statistical analysis**

Descriptive data will be presented as means with standard deviations, medians with range or frequencies, and percentages when appropriate. The non-parametric Mann-Whitney U test will be used to compare groups where data are skewed, for instance for PROM-results. A two-sided t-test will be used to analyse differences for normally distributed data. Categorical data will be analyzed using the Fisher exact test. We will conduct logarithmic transformation and linear regression or if more appropriate quartile regression, for multivariate regression using the generalized estimating equation model. Data will be analysed using the IBM SPSS Statistics and STATA. The significance level is set to 5%. Comparisons will also be done between different groups of stratification and the 25% of patients having the worst outcome in each treatment arm. All statistical analyses will be performed in cooperation with the statisticians at ØHT and NTNU. Reasons for ineligibility, non-compliance, withdrawal, or other protocol violations will be stated, and any patterns will be summarised. A more detailed statistical plan is available as *Supplementary file 2*.

**Funding**South-Eastern Norway Regional Health Authority has granted ØHT 50% employment of the PhD candidate for 6 years from August 2024. The research department of ØHT has guaranteed to cover costs for trial-related x-ray surveys, open access publishing and presenting our research at international conferences.

**Data safety and monitoring board (DSMB)**
Are H. Stødle, MD, PhD, consultant orthopaedic surgeon, Oslo University Hospital and Mette Andersen, MD, PhD, consultant orthopaedic surgeon, Aleris Hospital, Tromsø and Vestre Viken HT, have a mandate to stop the study if untoward effects are observed. Adverse events will be reported every 6 months and preliminary results from the first 30 patients 12 weeks follow up presented. Interim analysis of patient reported functional outcome and adverse events each treatment arms after 60 patients at follow up of 1 year.

**Ethics**

The Regional Committee for Medical and Health Research Ethics (REK Sørøst, ref. 496556) and the Data Protection Officers at all collaborating hospitals have approved patient recruitment to this trial. Participation is based on consent. Data collection and management will fulfil terms in approvals. Patients have been interviewed on the content in the PROMs and hence contribute to the development of Norwegian translations. Inger Lise Broen has taken part in our work as a user representative from the Consumer board of ØHT. She has given constructive feedback on layout and content on written patient information

**Potential harm**One can expect some side effects from one additional step in a surgical procedure. Side effects can be pain from the posterior tibial tendon or ankle stiffness or problems from the ankle or subtalar joint after deltoid ligament suture in case of malplacement of the anchor/ligament suture. Not yet being an everyday procedure, deltoid ligament repair might be challenging to perform for other than foot and ankle surgeons or experienced trauma surgeons. The protocol states that one of such surgeons are to take part in the surgical procedures. We do not find deltoid ligament repair difficult to teach. We believe that the moderate risk implied by this ligament suture may be outweighed by improved talar reduction, ankle stability and function. We will of course offer collaborating hospitals teaching in our procedure for deep deltoid ligament repair.

**Discussion**

The results from our trial will add some knowledge to the understanding of unstable ankle fractures and what treatment is necessary. Different options have been discussed before choosing treatment arms, also simply deltoid ligament repair without plating of the lateral malleolus, to turn a Lauge-Hansen SER4B into a SER2 fracture, a fracture that could be treated without surgery. This suggestion was found to be too controversial.

Another debate has been whether the ligament suture should be protected better than our minimum requirements of aftertreatment of two weeks in cast or walker boot. Orthosis is optional after two weeks. No further protection this early in the rehabilitation process may cause a loss of the potential effect of ligament suture.

During the planning of this trial, we became aware of differences in traditions between recruiting hospitals in choice of implants (anatomic or traditional 1/3 tube plates), buttons or syndesmotic screws and the threshold to use them or not. One hospital already used deep posterior deltoid ligament suture as the main supplement to osteosynthesis of the lateral malleolus. We know that a trans-syndesmotic fixation has a synergistic effect to deltoid ligament repair in stabilizing the ankle fork around the talus, and probably holding the torn deltoid ligament ends in proximity in most cases. Several of us have seen an ankle still being unstable after lateral plating, where the pathological medial talar tilt almost disappears after a trans-syndesmotic fixation.

We see the risk of trans-syndesmotic fixations hiding the potential effect of deep posterior deltoid ligament repair.

Nor do we know the effect of trans-syndesmotic fixation in a sample of SER4B fractures. This could have been a third arm of treatment. At the same time, we know that trans-syndesmotic fixations carry a high malreduction rate and the need of implant removal is quite frequent. When we were about to start the trial, realizing that established practice differs, we could not defend prohibiting the use of trans-syndesmotic implants in either group. The chosen arms of treatment attempts to be a pragmatic approach where the main difference between the groups will be whether the deep posterior deltoid ligament is repaired or not. Thereby we acknowledge that the solely plating treatment arm may contain several trans-syndesmotic fixations that may compensate for and conceal the true difference in outcome between additional deltoid ligament repair and solely plating. We are aware that the Lauge-Hansen classification does not predict extent of ligament injury precisely, and syndesmotic injury might be more important than fracture level of the fibula. Another weakness of our study is that it lacks a bilateral postoperative ankle CT scan of all patients, especially in cases where transsyndesmotic fixation is performed and malreposition is quite common. Since MRIs are not performed, we miss information about cartilage injuries that might affect patients’ outcomes.

**Disseminiation**

Results will be communicated to attending patients, presented in regional media and published in international peer-reviewed journals and presented at national and international conferences. Both positive and negative results will be reported, and co-authors will meet the criteria for co-authorship as defined by the International Committee of Medical Journal Editors.

Results from five years follow up will be published separately.

**6. References**

[1.    Berkowitz MJ, Sanders RW, Walling AK. Salvage arthrodesis after failed ankle replacement: surgical decision making. *Foot Ankle Clin*. 2012;17(4):725-740. doi:10.1016/j.fcl.2012.08.009](https://sciwheel.com/work/bibliography/17379713)

[2.    Brodke DJ, Saltzman CL, Brodke DS. PROMIS for orthopaedic outcomes measurement. *J Am Acad Orthop Surg*. 2016;24(11):744-749. doi:10.5435/JAAOS-D-15-00404](https://sciwheel.com/work/bibliography/8052745)

[3.    Butler BA, Hempen EC, Barbosa M, et al. Deltoid ligament repair reduces and stabilizes the talus in unstable ankle fractures. *J Orthop*. 2020;17:87-90. doi:10.1016/j.jor.2019.06.005](https://sciwheel.com/work/bibliography/16605925)

[4.    Campbell KJ, Michalski MP, Wilson KJ, et al. The ligament anatomy of the deltoid complex of the ankle: a qualitative and quantitative anatomical study. *J Bone Joint Surg Am*. 2014;96(8):e62. doi:10.2106/JBJS.M.00870](https://sciwheel.com/work/bibliography/508039)

[5.    Chan A-W, Boutron I, Hopewell S, et al. SPIRIT 2025 statement: updated guideline for protocols of randomised trials. *BMJ*. 2025;389:e081477. doi:10.1136/bmj-2024-081477](https://sciwheel.com/work/bibliography/18154596)

[6.    Chan A-W, Tetzlaff JM, Altman DG, et al. SPIRIT 2013 statement: defining standard protocol items for clinical trials. *Ann Intern Med*. 2013;158(3):200-207. doi:10.7326/0003-4819-158-3-201302050-00583](https://sciwheel.com/work/bibliography/594495)

[7.    Cöster MC, Bremander A, Rosengren BE, Magnusson H, Carlsson A, Karlsson MK. Validity, reliability, and responsiveness of the Self-reported Foot and Ankle Score (SEFAS) in forefoot, hindfoot, and ankle disorders. *Acta Orthop*. 2014;85(2):187-194. doi:10.3109/17453674.2014.889979](https://sciwheel.com/work/bibliography/2909783)

[8.    Cöster MC, Nilsdotter A, Brudin L, Bremander A. Minimally important change, measurement error, and responsiveness for the Self-Reported Foot and Ankle Score. *Acta Orthop*. 2017;88(3):300-304. doi:10.1080/17453674.2017.1293445](https://sciwheel.com/work/bibliography/12136194)

[9.    Dalen AF, Gregersen MG, Skrede AL, et al. Effects of progressive deltoid ligament sectioning on weber B ankle fracture stability. *Foot Ankle Int*. 2023;44(9):895-904. doi:10.1177/10711007231180212](https://sciwheel.com/work/bibliography/16606455)

[10.   Dawe EJC, Shafafy R, Quayle J, Gougoulias N, Wee A, Sakellariou A. The effect of different methods of stability assessment on fixation rate and complications in supination external rotation (SER) 2/4 ankle fractures. *Foot Ankle Surg*. 2015;21(2):86-90. doi:10.1016/j.fas.2014.09.010](https://sciwheel.com/work/bibliography/16605923)

[11.   Döring S, Provyn S, Marcelis S, et al. Ankle and midfoot ligaments: Ultrasound with anatomical correlation: A review. *Eur J Radiol*. 2018;107:216-226. doi:10.1016/j.ejrad.2018.08.011](https://sciwheel.com/work/bibliography/16605648)

[12.   Garratt AM, Naumann MG, Sigurdsen U, Utvåg SE, Stavem K. Evaluation of three patient reported outcome measures following operative fixation of closed ankle fractures. *BMC Musculoskelet Disord*. 2018;19(1):134. doi:10.1186/s12891-018-2051-5](https://sciwheel.com/work/bibliography/8509577)

[13.   Golanó P, Vega J, de Leeuw PAJ, et al. Anatomy of the ankle ligaments: a pictorial essay. *Knee Surg Sports Traumatol Arthrosc*. 2010;18(5):557-569. doi:10.1007/s00167-010-1100-x](https://sciwheel.com/work/bibliography/3071351)

[14.   Gougoulias N, Khanna A, Sakellariou A, Maffulli N. Supination-external rotation ankle fractures: stability a key issue. *Clin Orthop Relat Res*. 2010;468(1):243-251. doi:10.1007/s11999-009-0988-2](https://sciwheel.com/work/bibliography/8493016)

[15.   Gregersen MG, Dalen AF, Skrede AL, Bjelland Ø, Nilsen FA, Molund M. Effects of fibular plate fixation on ankle stability in a weber B fracture model with partial deltoid ligament sectioning. *Foot Ankle Int*. 2024;45(6):641-647. doi:10.1177/10711007241235903](https://sciwheel.com/work/bibliography/17016014)

[16.   Gregersen MG, Molund M. 2021 IFFAS Award for Excellence Winner: Weightbearing Radiographs Reliably Predict Normal Ankle Congruence in Weber B/SER2 and 4a Fractures: A Prospective Case-Control Study. *Foot & Ankle Orthopaedics*. 2022;7(1):2473011421S0002. doi:10.1177/2473011421S00029](https://sciwheel.com/work/bibliography/16605570)

[17.   Gustilo RB, Anderson JT. Prevention of infection in the treatment of one thousand and twenty-five open fractures of long bones: retrospective and prospective analyses. *J Bone Joint Surg Am*. 1976;58(4):453-458.](https://sciwheel.com/work/bibliography/3193922)

[18.   Gu G, Yu J, Huo Y, et al. Efficacy of deltoid ligament reconstruction on the  curative effect, complication and long-term prognosis  in ankle fracture-dislocation with deltoid ligament injury. *Int J Clin Exp Med*. 2017;2017;10:(9):13778-13783.](https://sciwheel.com/work/bibliography/17779599)

[19.   Hintermann B. Medial ankle instability. *Foot Ankle Clin*. 2003;8(4):723-738.](https://sciwheel.com/work/bibliography/5800833)

[20.   Jeong MS, Choi YS, Kim YJ, Kim JS, Young KW, Jung YY. Deltoid ligament in acute ankle injury: MR imaging analysis. *Skeletal Radiol*. 2014;43(5):655-663. doi:10.1007/s00256-014-1842-5](https://sciwheel.com/work/bibliography/6547876)

[21.   Jones CR, Nunley JA. Deltoid ligament repair versus syndesmotic fixation in bimalleolar equivalent ankle fractures. *J Orthop Trauma*. 2015;29(5):245-249. doi:10.1097/BOT.0000000000000220](https://sciwheel.com/work/bibliography/8486088)

[22.   Kellgren JH, Lawrence JS. Radiological assessment of osteo-arthrosis. *Ann Rheum Dis*. 1957;16(4):494-502. doi:10.1136/ard.16.4.494](https://sciwheel.com/work/bibliography/3806748)

[23.   Liao J, Zhang J, Ni W, Luo G. A retrospective study of deltoid ligament repair versus syndesmotic fixation in lateral malleolus fracture combined with both deltoid ligament injury and inferior tibiofibular syndesmotic disruption. *Front Surg*. 2022;9:912024. doi:10.3389/fsurg.2022.912024](https://sciwheel.com/work/bibliography/17778589)

[24.   Little MMT, Berkes MB, Schottel PC, et al. Anatomic fixation of supination external rotation type IV equivalent ankle fractures. *J Orthop Trauma*. 2015;29(5):250-255. doi:10.1097/BOT.0000000000000318](https://sciwheel.com/work/bibliography/16605927)

[25.   Mason LW, Kaye A, Widnall J, Redfern J, Molloy A. Posterior malleolar ankle fractures: an effort at improving outcomes. *JB JS Open Access*. 2019;4(2):e0058. doi:10.2106/JBJS.OA.18.00058](https://sciwheel.com/work/bibliography/16610910)

[26.   McKeown R, Parsons H, Ellard DR, Kearney RS. An evaluation of the measurement properties of the Olerud Molander Ankle Score in adults with an ankle fracture. *Physiotherapy*. 2021;112:1-8. doi:10.1016/j.physio.2021.03.015](https://sciwheel.com/work/bibliography/16606484)

[27.   Mittal R, Harris IA, Adie S, Naylor JM, CROSSBAT Study Group. Surgery for type B ankle fracture treatment: a combined randomised and observational study (CROSSBAT). *BMJ Open*. 2017;7(3):e013298. doi:10.1136/bmjopen-2016-013298](https://sciwheel.com/work/bibliography/16605604)

[28.   Mococain P, Bejarano-Pineda L, Glisson R, et al. Biomechanical effect on joint stability of including deltoid ligament repair in an ankle fracture soft tissue injury model with deltoid and syndesmotic disruption. *Foot Ankle Int*. 2020;41(9):1158-1164. doi:10.1177/1071100720929007](https://sciwheel.com/work/bibliography/11669716)

[29.   Molund M, Hellesnes J, Berdal G, Andreassen BS, Andreassen GS. Compared to conventional physiotherapy, does the use of an ankle trainer device after Weber B ankle fracture operation improve outcome and shorten hospital stay? A randomized controlled trial. *Clin Rehabil*. 2020;34(8):1040-1047. doi:10.1177/0269215520929727](https://sciwheel.com/work/bibliography/16606442)

[30.   Naumann MG, Sigurdsen U, Utvåg SE, Stavem K. Functional outcomes following surgical-site infections after operative fixation of closed ankle fractures. *Foot Ankle Surg*. 2017;23(4):311-316. doi:10.1016/j.fas.2016.10.002](https://sciwheel.com/work/bibliography/16606459)

[31.   Olerud C, Molander H. A scoring scale for symptom evaluation after ankle fracture. *Arch Orthop Trauma Surg*. 1984;103(3):190-194. doi:10.1007/BF00435553](https://sciwheel.com/work/bibliography/11398418)

[32.   Pakarinen HJ, Flinkkilä TE, Ohtonen PP, et al. Syndesmotic fixation in supination-external rotation ankle fractures: a prospective randomized study. *Foot Ankle Int*. 2011;32(12):1103-1109. doi:10.3113/FAI.2011.1103](https://sciwheel.com/work/bibliography/5238703)

[33.   Park YH, Jang KS, Yeo ED, Choi GW, Kim HJ. Comparison of outcome of deltoid ligament repair according to location of suture anchors in rotational ankle fracture. *Foot Ankle Int*. 2021;42(1):62-68. doi:10.1177/1071100720952053](https://sciwheel.com/work/bibliography/16966208)

[34.   Paulsen A, Djuv A, Garratt AM, Nguyen MQ. OLERUD-MOLANDER ANKLE SCORE: EN NORSK OVERSETTELSE OG KRYSSKULTURELL ADAPTASJON. In: Nof; 2022:84.](https://sciwheel.com/work/bibliography/17204320)

[35.   Rabin R, de Charro F. EQ-5D: a measure of health status from the EuroQol Group. *Ann Med*. 2001;33(5):337-343. doi:10.3109/07853890109002087](https://sciwheel.com/work/bibliography/1175040)

[36.   Salameh M, Alhammoud A, Alkhatib N, et al. Outcome of primary deltoid ligament repair in acute ankle fractures: a meta-analysis of comparative studies. *Int Orthop*. 2020;44(2):341-347. doi:10.1007/s00264-019-04416-9](https://sciwheel.com/work/bibliography/16966498)

[37.   Schock HJ, Pinzur M, Manion L, Stover M. The use of gravity or manual-stress radiographs in the assessment of supination-external rotation fractures of the ankle. *J Bone Joint Surg Br*. 2007;89(8):1055-1059. doi:10.1302/0301-620X.89B8.19134](https://sciwheel.com/work/bibliography/13145447)

[38.   Seidel A, Krause F, Weber M. Weightbearing vs Gravity Stress Radiographs for Stability Evaluation of Supination-External Rotation Fractures of the Ankle. *Foot Ankle Int*. 2017;38(7):736-744. doi:10.1177/1071100717702589](https://sciwheel.com/work/bibliography/8486400)

[39.   Sogbein OA, Saad L, Barton KI, et al. High-Energy Transsyndesmotic Ankle Fracture Dislocation-Does Deltoid Ligament Repair Influence the Progression of Posttraumatic Arthritis in Logsplitter Injuries? *J Orthop Trauma*. 2024;38(11):615-621. doi:10.1097/BOT.0000000000002877](https://sciwheel.com/work/bibliography/17402262)

[40.   Strömsöe K, Höqevold HE, Skjeldal S, Alho A. The repair of a ruptured deltoid ligament is not necessary in ankle fractures. *J Bone Joint Surg Br*. 1995;77(6):920-921. doi:10.1302/0301-620X.77B6.7593106](https://sciwheel.com/work/bibliography/16605624)

[41.   Stufkens SAS, van den Bekerom MPJ, Kerkhoffs GMMJ, Hintermann B, van Dijk CN. Long-term outcome after 1822 operatively treated ankle fractures: a systematic review of the literature. *Injury*. 2011;42(2):119-127. doi:10.1016/j.injury.2010.04.006](https://sciwheel.com/work/bibliography/6894833)

[42.   Wang J, Stride D, Horner NS, et al. The role of deltoid ligament repair in ankle fractures with syndesmotic instability: A systematic review. *J Foot Ankle Surg*. 2021;60(1):132-139. doi:10.1053/j.jfas.2020.02.014](https://sciwheel.com/work/bibliography/15757907)

[43.   Weber M, Burmeister H, Flueckiger G, Krause FG. The use of weightbearing radiographs to assess the stability  of supination-external rotation fractures of the ankle. *Arch Orthop Trauma Surg*. 2010;130(5):693-698. doi:10.1007/s00402-010-1051-1](https://sciwheel.com/work/bibliography/16605935)

[44.   Whitlock KG, LaRose M, Barber H, et al. Deltoid ligament repair versus trans-syndesmotic fixation for bimalleolar equivalent ankle fractures. *Injury*. 2022;53(6):2292-2296. doi:10.1016/j.injury.2022.03.063](https://sciwheel.com/work/bibliography/17402252)

[45.   Wu K, Lin J, Huang J, Wang Q. Evaluation of transsyndesmotic fixation and primary deltoid ligament repair in ankle fractures with suspected combined deltoid ligament injury. *J Foot Ankle Surg*. 2018;57(4):694-700. doi:10.1053/j.jfas.2017.12.007](https://sciwheel.com/work/bibliography/16605596)

[46.   Zhao H-M, Lu J, Zhang F, et al. Surgical treatment of ankle fracture with or without deltoid ligament repair: a comparative study. *BMC Musculoskelet Disord*. 2017;18(1):543. doi:10.1186/s12891-017-1907-4](https://sciwheel.com/work/bibliography/17778579)

[47.   Zhong B. How to calculate sample size in randomized controlled trial? *J Thorac Dis*. 2009;1(1):51-54.](https://sciwheel.com/work/bibliography/2790916)
