# Supplementary material for: The Benefit of Repairing the Deltoid Ligament in Unstable Ankle Fractures: Patient-Reported Functional Outcome and Radiological Stability Measurements; a Clinical Trial Protocol
Source: Foot Ankle Orthop. 2025 Nov 12;10(4):24730114251386735. doi: 10.1177/24730114251386735 (PMC12615956; doi:10.1177/24730114251386735)
Supplement: sj-docx-6-fao-10.1177_24730114251386735 – Supplemental material for The Benefit of Repairing the Deltoid Ligament in Unstable Ankle Fractures: Patient-Reported Functional Outcome and Radiological Stability Measurements; a Clinical Trial Protocol [file sj-docx-6-fao-10.1177_24730114251386735.docx]

This Statistical Analysis Plan (SAP) follows the “Guidelines for the Content of Statistical Analysis Plans in Clinical Trials”, complying with the ICH E9 guideline.

Administrative information:

| Sponsor name | South-Eastern Norway Regional Health Authority  Østfold Hospital Trust |
| --- | --- |
| Sponsor address | Parkgata 36 N-2302 Hamar  Kalnesveien 300  N-1714 Grålum |
| REC no | The Regional Committee for Medical and Health Research Ethics ref. #496556 The Data Protection Officers at all collaborating hospitals have approved patient recruitment to this trial. |
| Trial title | “The benefit of repairing the deltoid ligament in unstable ankle fractures. Patient-reported functional outcome and radiological stability measurements.”  A randomized controlled study to evaluate whether additional deltoid ligament repair preserves function better than the established treatment with lateral fracture repair and sometimes a trans-syndesmotic fixation. |
| Trial ID | “Deltoidligamentstudien” 2024104 |
| Trial registration number | ClinicalTrials.gov NCT2024104 |

SAP and protocol version:

| SAP version and date: | This SAP is version 2, dated 23^rd^ of January 2025 |
| --- | --- |
| Protocol version | This document refers to the Study protocol version 2, publishing version, dated 23^rd^ of January 2025 |

**SAP revision history:**

The main Statistic Analysis Plan has been unchanged through the planning process from the time our Protocol was approved by the REC in 2022. Some additional analysis have been planned, like comparing the tails between the allocated groups; the 25% of patients having the worst outcome in each treatment arm

What shall be reported to the Data Safety and Monitoring Board (DSMB) and interim analyses that shall be presented to them has been specified before inclusion was started in September 2024.

After Gamble et al *(JAMA 2017).*

**ABBREVIATIONS**

| AE | Adverse Event |
| --- | --- |
| BMI | Body Mass Index |
| CI | Confidence Interval |
| SD | Standard Deviation |
| DSMB | Data Safety and Monitoring Board |

**SIGNATURE PAGE**

|  | **PRINCIPAL/COORDINATING INVESTIGATOR:** |  |  |
| --- | --- | --- | --- |
|  | Name, title: Prof. Dr. Med Frede Jon Frihagen, MD, PhD  Affiliation: Østfold Hospital Trust, Kalnes University of Oslo |  |  |
|  |  |  |  |
|  | Signature |  | 23/01/2025 |
|  |  |  |  |
|  | **MAIN CHIEF INVESTIGATOR:** |  |  |
|  | Name, title: Dr. Marius Molund, Head of Department of Orthopaedic Surgery  Affiliation: Østfold Hospital Trust |  |  |
|  |  |  |  |
|  | Signature |  | 23/01/2025 |
|  |  |  |  |
|  |  |  |  |

**TABLE OF CONTENTS**

[1 Introduction 5](#_Toc206152994)

[1.1 Background and Rationale 5](#_Toc206152995)

[1.2 Intervention(s) 5](#_Toc206152996)

[1.3 Trial Objectives 5](#_Toc206152997)

[2 Trial Methods 5](#_Toc206152998)

[2.1 Trial Design 5](#_Toc206152999)

[2.2 Randomization 5](#_Toc206153000)

[2.3 Statistical Framework 6](#_Toc206153001)

[2.4 Timing of Outcome Assessments 7](#_Toc206153002)

[2.5 Statistical Interim Analyses and Stopping Guidance 8](#_Toc206153003)

[2.6 Timing of Main Analysis 8](#_Toc206153004)

[3 Trial Population 8](#_Toc206153005)

[3.1 Screening Data, Eligibility and Recruitment 8](#_Toc206153006)

[3.2 Baseline Patient Characteristics 9](#_Toc206153007)

[3.3 Withdrawal/Follow-up 9](#_Toc206153008)

[3.4 Adherence and Protocol Deviations 10](#_Toc206153009)

[3.5 Analysis Populations 10](#_Toc206153010)

[4 Outcome Definitions 11](#_Toc206153011)

[4.1 General Definitions and Derived Variables 11](#_Toc206153012)

[4.2 Primary Outcome Definition 11](#_Toc206153013)

[4.3 Secondary Outcome Definition 11](#_Toc206153014)

[5 Analysis Methods 11](#_Toc206153015)

[5.1 Methods for Primary Outcome 11](#_Toc206153016)

[5.2 Methods for Dichotomous Secondary Outcomes 12](#_Toc206153017)

[5.3 Methods for Continuous Secondary Outcomes 13](#_Toc206153018)

[5.4 Additional Analyses 14](#_Toc206153019)

[5.5 Sample size 14](#_Toc206153020)

[6 Statistical Software 14](#_Toc206153021)

[7 References 14](#_Toc206153022)

[7.1 Literature References 14](#_Toc206153023)

[7.2 Reference to Data Handling Plan Link to the data management plan: 15](#_Toc206153024)

[7.3 Reference to the Trial Master File and Statistical Documentation 15](#_Toc206153025)

[7.4 Reference to other Standard Operating Procedures or Documents 15](#_Toc206153026)

# Introduction

## Background and Rationale

A major number of ankle fractures treated in former study samples presenting outcome after surgical treatment were less severe and would be treated conservatively with current guidelines. This supports the need for studies on fractures still chosen for surgery with current guidelines, a group of more severe fractures than in the samples most research has been done on until now.

Our trial may give crucial knowledge on whether additional deltoid ligament repair preserves function better than the established treatment with lateral fracture repair and sometimes a trans-syndesmotic fixation**.**

## Intervention(s)

### Brief description of the study intervention(s)

Surgical treatment should be performed by equally experienced surgeons in both the intervention and comparator groups. Fellowship-trained foot and ankle or trauma surgeons with lower extremity experience. Surgical procedures should be performed within 2 weeks after injury. Aftertreatment protocol is equal for both treatment arms, I refer to §2.4 Figure 1.

## Trial Objectives

### Primary Objective

In our trial we will test whether additional deltoid ligament repair preserves function better than the established treatment with lateral fracture repair and sometimes a trans-syndesmotic fixation**.**

### Secondary Objectives

We will also investigate whether deltoid ligament repair improves restoration of medial ankle joint congruency and deltoid ligament patency. And to what extent this extra procedure affects duration of surgery, perioperative complications and the progress of rehabilitation. We also want to know whether this can help us avoiding post-fracture arthritis in the long run, probably beyond the scope of our two-year follow-up, and maybe also beyond five years.

# Trial Methods

## Trial Design

Multicentre randomized controlled non-blinded trial.

## Randomization

Eligible patients are allocated in a 1:1 ratio to either solely plating of the lateral fracture or additional deltoid ligament repair. Stratification for block randomisation will be by biological sex and age above or below 50 years at the time of inclusion.

## Statistical Framework

### Hypothesis Test

This trial is designed to investigate whether additional deltoid ligament suture is superior to solely plating of the lateral malleolus when taking minimal important difference (MID) into consideration.

Primary null hypothesis: «Patient-reported function in Olerud Molander Ankle Score (OMAS) score after additional deltoid ligament suture will not be clinically superior to after only plating of lateral malleolar fractures in Lauge Hansen SER 4b fractures one and two years after surgery»

Primary alternative hypothesis: «Patient-reported function in Olerud Molander Ankle Score (OMAS) score after additional deltoid ligament suture is clinically superior to after only plating of lateral malleolar fractures in Lauge Hansen SER 4b fractures one and two years after surgery»

The primary hypothesis will also be tested for patient-reported function in A-FORM and SEFAS as they give more nuanced responses and have a less categorical scoring system than the OMAS.

Secondary null hypotheses: “The difference in medial clear space measurements of injured compared to non-injured side on weightbearing x-rays will not be significantly different between groups” and

“The difference between groups in medial clear space change measured in mm from weightbearing x-rays to gravity stress images of injured side will not be statistically significant at one or two years”.

Secondary alternative hypotheses: “The difference in medial clear space measurements of injured compared to non-injured side on weightbearing x-rays is significantly different between groups” and

“The difference between groups in medial clear space change measured in mm from weightbearing x-rays to gravity stress images of injured side is statistically significant at one or two years”.

A significant difference of medial clear space is set to be >1,0 mm.

### Confidence Intervals and p-values

All calculated p-values will be two-sided and compared to a 5% significance level. If a p-value is less than 0.05, the corresponding treatment group difference will be denoted as statistically significant. All efficacy estimates will be presented with two-sided 95% confidence intervals. As there is one single primary null hypothesis to be tested in this trial, there will be no adjustments for multiplicity.

### Decision Rule

This trial is designed to address one single primary outcome. Superiority is claimed if the primary null hypothesis is rejected on the significance level (alpha) of 0.05 (two-sided).

There are secondary endpoints that may be as important as the main end point, the other PROM scores Ankle Fracture Outcome of Rehabilitation Measure (A-FORM) Self-Reported Foot and Ankle Score (SEFAS) will also be presented while evaluating the main results.

## Timing of Outcome Assessments

I refer to the SPIRIT 2025 diagram also copied into the SPIRIT checklist.

| **Figure 1 \| SPIRIT 2025 diagram of the schedule of enrolment, interventions, and assessments** | | | | | |
| --- | --- | --- | --- | --- | --- |
| **Time point** |  | | | | |
| **Enrolment** | Screening for eligibility | | Refer to Table 1 eligibility criteriae. | | |
|  | Informed consent | | Written and oral information from a physiotherapist, nurse or orthopaedic surgeon other than the operating. | | |
|  | Randomization | | Randomization module in the eFORSK database (Block) | | |
|  | | | | | |
| **Surgical intervention**  (within 2 weeks after injury) | Intervention  *Lat. plating only* | | Surgical treatment should be performed by equally experienced surgeons in both the intervention and comparator groups. Fellowship-trained foot and ankle or trauma surgeons with lower extremity experience.  Observations from hospital stay and operation: Injury and repair description, duration of surgery. Per- and perioperative complications, length of hospital admission. | | |
|  | Comparator  *Deltoid lig. repair* | |  |  |  |
|  | | | | | |
| **Rehabilitation protocol** | Equal for both arms | | Up to two weeks in cast or Walker-orthosis and partial weight-bearing 20 kg. Movement exercises and weight-bearing as tolerated. Orthosis optional from two weeks. | | |
|  | | | | | |
| **Assessments** | | | | | |
| **Time of follow-up** | **WBXR*** | **Gravity stress XR** | | **PROM** (e-survey) | **Other** |
| 6 weeks ± 10 days | X |  | |  | VAS pain, adverse events. Use of orthosis and supporting aids. |
| 12 weeks ± 2 w | X |  | | X | As for 6w. Time of return to work? |
| 1 year ± 1 month | X | X | | X** | Validation of test-retest properties |
| 2 years ± 2 m | X |  | | X | Last observation for main publication |
| 5 years ± 5 m | X | X | | X | Will be published in a later paper |

*Weight-bearing x-rays (WBXR) of both legs in the mortise projection and measurement of MCS at all times of follow-up.
**sent twice for test-retest validation of the PROMs used in Norwegian versions, in particular the Ankle Fracture Outcome of Rehabilitation Measure (A-FORM) translation.
Gravity stress x-rays (after Michelson, *Clin Orthop Relat Res.* 2001 and Schock et al, *JBJS(Br)* 2007) performed at 1- and 5-years follow-up for evaluation of superficial deltoid ligament integrity at one and five years). Adverse events as peroperative complications, thrombosis, surgical site infection, other postoperative complications and reoperations are registered. Posttraumatic arthritis is registered according to the Kellgren and Lawrence scale.

## Statistical Interim Analyses and Stopping Guidance

The Data Safety and Monitoring Board have a mandate to stop the study if untoward effects are observed. Adverse events will be reported every 6 months, and preliminary results from the first 30 patients 12 weeks follow up presented. Interim analysis of patient-reported functional outcome and adverse events each treatment arm after 60 patients at follow-up of 1 year.

## Timing of Main Analysis

I refer to §2.4, Figure 1 SPIRIT 2025 schedule

# Trial Population

## Screening Data, Eligibility and Recruitment

**Table 1. Eligibility criteria.**

| Patients are eligible if they present to one of the participating hospitals and comply with the inclusion and exclusion criteria. | |
| --- | --- |
| **Inclusion criteria** 18-65 years of age at presentation | **Exclusion criteria** |
| Initial medial clear space (MCS) ≥7mm or weightbearing x-ray evaluated as unstable (side to side difference >1 mm ***or*** | Assumed not compliant (drug use, cognitive- and/or psychiatric disorders). |
| Fracture dislocation (*when doubt about state prior to reduction shall WBXR be performed*) | Insufficient language skills (Scandinavian) |
| Able to walk without aids before the injury | Multi-trauma or pathologic fracture |
| Posterior malleolus fragment Mason & Molloy 1 or no posterior malleolus fragment[^15^](https://sciwheel.com/work/citation?ids=16610910&pre=&suf=&sa=0&dbf=0) | Neuropathies and symptomatic generalized joint disease such as Rheumatoid Arthritis |
| Surgery planned within 2 weeks after injury and available for follow up | Previous ipsilateral former ankle surgery or fracture or previous injury with marked sequela of the lower limb |
| No syndesmotic screw or suture button planned prior to the surgical procedure | Open fx Gustilo-Anderson II or more[^23^](https://sciwheel.com/work/citation?ids=3193922&pre=&suf=&sa=0&dbf=0) or other medial soft tissue problem considerably increasing risk of additional medial approach to the ankle. |
| No other more severe condition in the same extremity |  |

A CONSORT flow diagram (appendix A) will be used to summarise the number of patients who were:

- assessed for eligibility at screening
- eligible at screening
- ineligible at screening*
- eligible and randomised
- eligible but not randomised*
- received the randomised allocation
- did not receive the randomised allocation*
- lost to follow-up*
- cross-over between allocations*
- randomised and included in the primary analysis
- randomised and excluded from the primary analysis*

*Reasons will be provided.

## Baseline Patient Characteristics

The patient demographics and baseline characteristics to be summarised include sex, age in years and BMI and smoking status. If the fracture is open or closed according to the Gustilo Anderson-classification and if there is a posterior malleolar fragment or Chaput-tillaux fragment will also be registered.

Descriptive data will be presented in a table as means with standard deviations, medians with range or frequencies, and percentages when appropriate.

Any clinical important imbalance between the treatment groups will be noted.

For presentation I refer to §5

## Withdrawal/Follow-up

Patients attending the trial may withdraw from follow-up and withdraw consent for data collected to date to be used; or be lost to contact/follow-up. Patients are informed that withdrawal must be claimed before statistical analysis is performed.

The status of eligible and randomized patients at trial end will be tabulated by treatment group according to

- completed intervention and assessments
- withdrew consent
- lost to follow-up, and at what stage (after 12 weeks, 1, 2 or 5 years)

Timing of withdrawals will be included in the CONSORT flow diagram. For each follow-up time point information on the number of withdrawals and reasons for withdrawal, number included in the analysis and the number died (if applicable) will be reported.

## Adherence and Protocol Deviations

### Adherence to Allocated Treatment

As the intervention is the surgical procedure performed shortly after randomization, we consider the patients to be adherent if the main procedures performed in case of a reoperation are of the same kind as the patient was allocated to. Deltoid ligament repair for a patient allocated to lateral plating only means a cross-over.

### Protocol Deviations

A protocol deviation is defined as a failure to adhere to the protocol such as the wrong intervention being administered, incorrect data being collected and documented, errors in applying inclusion/exclusion criteria or missed follow-up visits.

The following are pre-defined major protocol deviations regarded to affect the efficacy of the intervention:

- Entering the trial when the eligibility criteria should have prevented trial entry
- Received other intervention than allocated

The number (and percentage) of patients with major and minor protocol deviations will be summarised by treatment group with details of type of deviation provided. Comparison with T-tests for major protocol deviations is not supposed to show significant differences as major protocol deviations are expected to be rare.

We expect minor protocol deviations as the surgical technique instruction states that: “If the surgeon notices severe instability that he or her finds compulsory to address further, this should be performed, and actions noted in the protocol.” This is expected to imply for instance the use of syndesmotic screws or buttons and repair of other structures. The prevalence of such efforts in each group may affect the results and will be investigated by two-sided T-tests.

## Analysis Populations

The Enrolled set will include all patients who have provided informed consent and have been included into the study data base.

The Per Protocol Analysis Set (PPS) will include all randomized patients meeting the study eligibility criteria and with no major protocol deviations affecting the treatment efficacy.

# Outcome Definitions

## General Definitions and Derived Variables

### Body Mass Index

Body Mass Index (BMI) = Body weight in kilograms divided by the square of the height in meters.

## Primary Outcome Definition

Primary outcome: Patient-reported function in Olerud-Molander Ankle Score (OMAS) after lateral malleolar plating only versus additional deltoid ligament suture at 1 and 2 years. Results at 12 weeks will also be reported. The primary outcome is continous. The minimal clinical important difference for the OMAS is found to be 9,7 out of 100 points (McKeown & al, *Physiotherapy 2021*)

## Secondary Outcome Definition

Secondary outcomes:
-Infection, reoperation and other major adverse events shall be registered and compared between groups.

-Difference in medial clear space on WBXRs or gravity stress images after surgery with or without deltoid ligament repair. Sectra Picture Archiving and Communications System (Sectra AB, Linköping, Sweden) will be used for radiographic measurements.

-Signs of post-fracture arthritis is another important outcome and will be reported according to Kellgren Lawrence Scale.

-Function reported by Ankle Fracture Outcome of Rehabilitation Measure (A-FORM) Self-Reported Foot and Ankle Score (SEFAS) Patient-Reported Outcomes Measurement Information System (PROMIS), Visual Analogue Scale (VAS) pain and Euroquol EQ-5D 5L. The former is generic; referring to the individuals general health, OMAS and the other named Patient Reported Outcome Measures (PROMs) are specific to the anatomic region.

-For 1 year follow up we added a 7-point Likert scale about change in function after 12 weeks to use as an anchor for comparison with change in PROM scores and responsiveness calculations.

In 1- and 2- and 5-years follow-up we supplemented with the patient acceptable symptom state (PASS) value “Taking into account all the activities you have during your daily life, your level of pain, and also your functional impairment, do you consider that your current state is satisfactory?” (yes/no)

# Analysis Methods

## Methods for Primary Outcome

For primary outcomes I refer to §2.4 Figure 1.

We assume that PROM-results are not normally distributed. PROM scores are continuous variables.

Groups will be compared using the non-parametric Mann-Whitney U-test (Wilcoxon rank sum test).

The 25 per cent of patients in each treatment arm having the worst outcome will be compared using the same test.

Comparisons will also be done between different groups of stratification (biological sex and age above or below 50 years).

### Descriptive Statistics

Descriptive statistics will include number and percentage by treatment group. Descriptive statistics will be based on non-imputed data, thus the number of evaluable outcome measurements at the time of primary interest (52 weeks) will also be presented.

### Missing Data

For the primary outcome, missing data occur if a patient does not attend follow-up visits of 12 weeks, 1 or 2 years or answer their related electronical PROM-surveys.

No imputation will be done for missing data, patients not reporting will be left out of the analysis.

1. Restricting the primary analysis to the PPS

## Methods for Dichotomous Secondary Outcomes

Main findings:

Loss to follow-up and crossovers will be registered.

Main implant used for lateral malleolar fixation:
 -anatomic plate (with angular stability) or classic simple tubular plate

The use of additional fixation procedures
 -syndesmotic screw or button
 -fixation of posterior malleolus or Chaput-Tillaux fragments
 -direct suture of ligaments other than the deltoid ligament
 -other
 -peroperative complications?

Exploratory findings of interest:

| **Patients allocated to deltoid ligament repair: injury and repair description** | | | |
| --- | --- | --- | --- |
| Tear | None | Partial | Complete |
| Site of tear of deep posterior component | Medial malleolus posterior colliculus | Midsubstance | Talus |
| Placement of posterior anchor | Medial malleolus posterior colliculus | No posterior anchor | Talus |

Reoperations will be registered and indication specified as implant removal during the time of follow up, deep infection or wound dehiscence or fixation failure or other reason (to be described in surgery record)

Arthritis graded Kellgren Lawrence 3 or 4 with joint space narrowing will be registered as a dichotomous outcome

PASS-value answer (yes/no)

Other findings: listed in a table

| Use of temporary external fixator |
| --- |
| Findings from tests for deltoid integrity (talar shift and tilt tests) |
| Findings from syndesmotic-specific tests |
| Main kind of anaesthesia |
| Nerve block administered? |
| Torniquet peroperatively? |
| Use of anticoagulant or platelet inhibiting drugs |
| Deep venous thrombosis (pulmonary embolism specified in addition) |
| Delayed wound healing other than deep infection (not dry at 2w?) |
| Able to bear full weight at 6 w? |
| How many weeks in orthosis |
| How many weeks using other supporting aids? |
| How many weeks before return to work? (Same as earlier?) |

The Fisher exact test will be used for comparison of categorical data.

## Methods for Continuous Secondary Outcomes

For secondary outcomes I refer to §2.4 Figure 1 for the ankle specific PROMs A-FORM and SEFAS. Differences between groups in PROM-results will be assessed using the Mann Whitney U-test (Wilcoxon rank sum test) and results presented in box plots for each of the PROMs mentioned. Surgery duration will be presented in a table comparing group results with means and SDs together with PROM-scores including EQ5D-5L and VAS Pain

Days of hospital stay for groups will be reported.

## Additional Analyses

Post-hoc power calculations will be performed based on the MCID calculated from our trial sample for the respective PROMs used. We will evaluate their measurement properties including test-retest reliability and responsiveness by anchor-based methods. This validation study will use change in function (PROM) from 12 weeks to 1 year compared with a 7-point Likert scale, and test-retest data from repetition of our survey after 10 days at 1 year follow-up.

Regression analysis will be performed to examine for correlation between groups of randomization, variables of stratification (sex and age above or under 50 years), additional surgical procedures (mainly trans-syndesmotic screws or buttons), function in PROM and medial clear space differences.

As we presume that the data (outcome scores) are not normally distributed, logarithmic transformation will be performed before linear regression for the comparison of single assessments. Median regression and Poisson-regression will be used exploratory. If linear regression after transformation shows not to be adequate, quartile regression will be used. The coefficients found and interpreted will be compared.

For comparison of the overall assessments, we will conduct multivariate regression using the generalized estimating equation models. Incidence of post-fracture arthritis is of great interest relative to the mentioned possible covariates and will also be assessed but will presumably be more relevant at the 5-years follow-up.

## Sample size

Sample size calculation has been based on the primary endpoint patient-reported function in OMAS. The former defined minimal clinically important difference of between 8 and 10 (slightly more than half of the standard deviation (SD), or 9,7 as referred by McKeown et al (*Physiotherapy 2021*)) in Olerud-Molander ankle score between the study-groups, expected SD 14 points (Pakarinen (*FAI 2011*) et al found 13 and 15 in their groups. Molund et al had a SD of 12 on average (*Clin Rehabil. 2020*), significance level *α* = 0*.*05, Power 0,8, and estimated 20% drop out (two-sided t-test, clinical superiority design) we will include 60 patients in each group to ensure sufficient power after expected drop out of 20% (Zhong et al, *J Thorac Dis. 2009*)*.*

# Statistical Software

All statistical analyses will be done using the IBM SPSS Statistics and STATA (StataCorp. 2015. *Stata Statistical Software: Release 14*. College Station, TX, USA).

# References

## Literature References

1. Guidelines for the Content of Statistical Analysis Plans in Clinical Trials. 2017;318(23):2337-2343. doi:10.1001/jama.2017.18556.
2. Zhong B. How to calculate sample size in randomized controlled trial? J Thorac Dis. 2009;1(1):51-54.
3. Rosner B. Essentials of Biostatistics. 8^th^ edition 2016. CENGAGE Learning.
4. The template for this Statistical Analysis Plan is picked from [www.norcrin.no](http://www.norcrin.no) (Metodebok.no)

## Reference to Data Handling Plan Link to the data management plan: <https://dmp.sikt.no/plan/8678ef77-fad1-4bdf-8a74-3c07341ede2c>

## Reference to the Trial Master File and Statistical Documentation

ClinicalTrials.gov # 2024104

## Reference to other Standard Operating Procedures or Documents

**The SPIRIT checklist 2025 “Deltoidligamentstudien” is linked at the page**

<https://www.sykehuset-ostfold.no/kliniske-studier/skal-vi-reparere-deltoidligamentet-ved-ustabile-ankelbrudd-funksjon-rapportert-av-pasienter-etter-operativ-behandling.-stabilitetsmal-pa-rontgenbilder>

Appendix 1


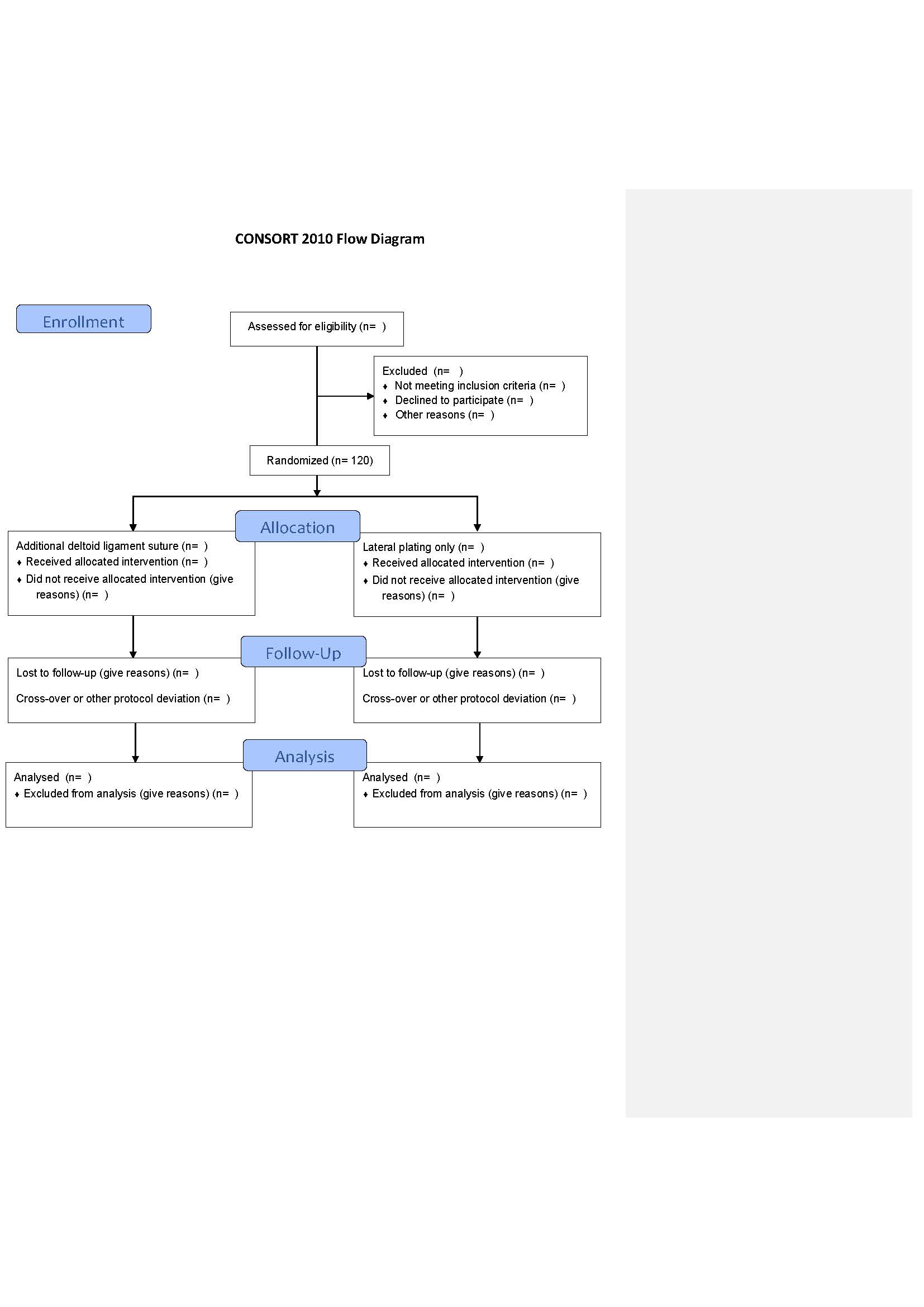


CC.BY-4.0

Inclusion criteria: Weber B fractures with full tear of the deltoid ligament (SER4B.)

- Initial medial clear space (MCS)>=7mm or Weightbearing X-Ray (WBXR) evaluated as unstable or
- Fracture dislocation (*when doubt about state prior to reposition: WBXR)*
- 18-65 years of age at presentation
- Able to walk without aids before the injury.
- Posterior malleolus fragment Mason & Molloy 1 or no posterior malleolar fragment
- No more severe condition in the same extremity
- Surgery planned within 2 weeks after injury and available for follow-up
- No syndesmotic screw or suture button planned prior to the surgical procedure

Exclusion criteria Assumed not compliant (drug use, cognitive and/or psychiatric

disorders).

- Insufficient language skills (Scandinavian)
- Former ankle surgery or significant sequela of injury to the lower limb
- Open fx Gustilo-Anderson II or more or medial soft tissue state considerably increasing risk of a medial ankle approach.
- Multi-trauma and pathologic fracture.
- Neuropathies and symptomatic generalized joint disease such as Rheumatoid Arthritis
