# Supplementary material for: The Benefit of Repairing the Deltoid Ligament in Unstable Ankle Fractures: Patient-Reported Functional Outcome and Radiological Stability Measurements; a Clinical Trial Protocol
Source: Foot Ankle Orthop. 2025 Nov 12;10(4):24730114251386735. doi: 10.1177/24730114251386735 (PMC12615956; doi:10.1177/24730114251386735)
Supplement: sj-pdf-2-fao-10.1177_24730114251386735 – Supplemental material for The Benefit of Repairing the Deltoid Ligament in Unstable Ankle Fractures: Patient-Reported Functional Outcome and Radiological Stability Measurements; a Clinical Trial Protocol [file sj-pdf-2-fao-10.1177_24730114251386735.pdf]

Deltoid ligament repair technique

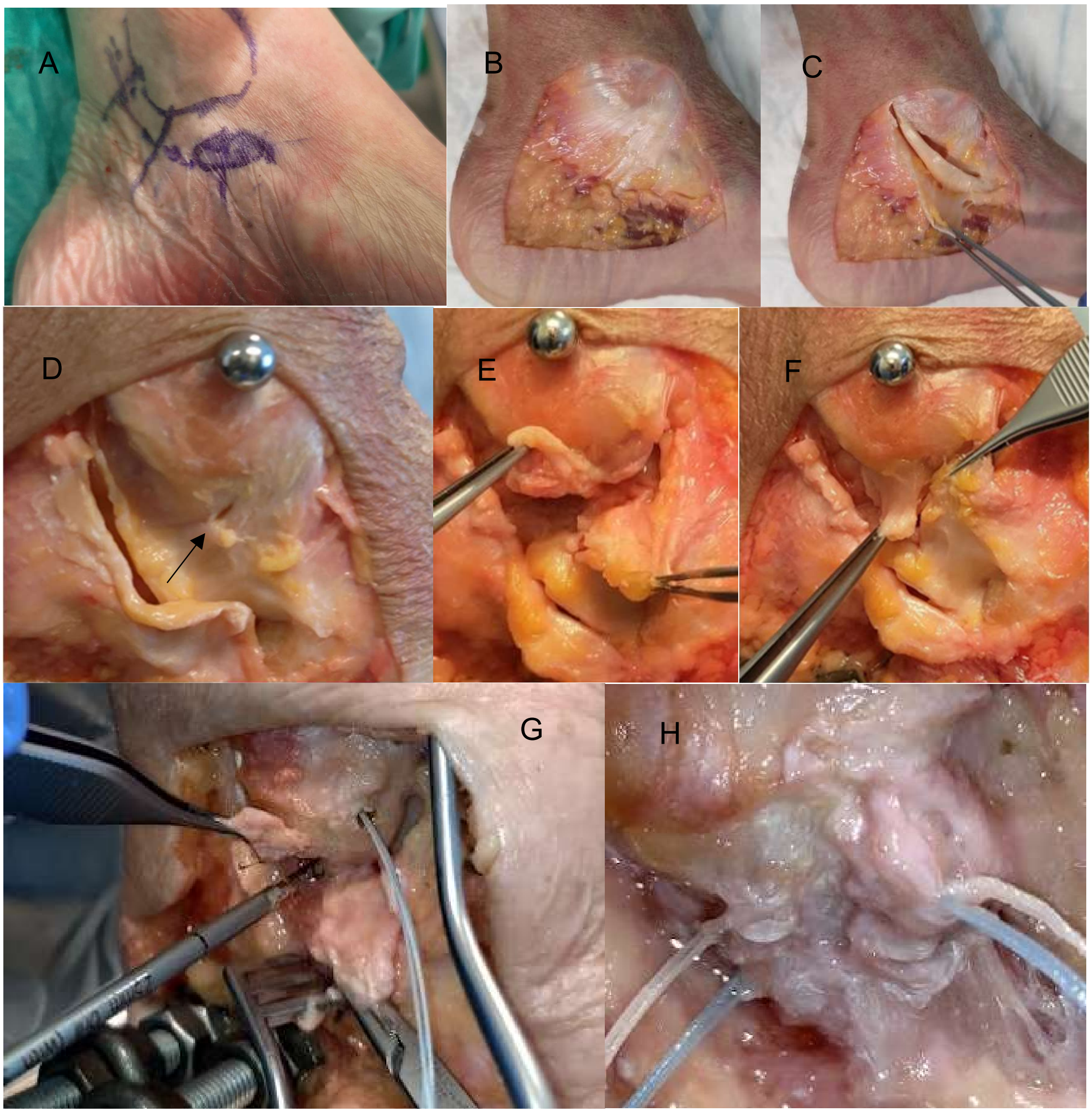

A: Topographic orientation medial malleolus and sustentaculum tali. Identification of the os naviculare more anteriorly may also be helpful.

B: Notice retinacular structures overlying the tibialis posterior tendon.

C. Tendon retinaculum incised close to the posterior tibia and medial malleolus.

D: Cadaver dissected and marked with a metal calibration bullet in the distal tibia. Arrow aiming at the chosen interval between major anterior and posterior elements of the deltoid ligament.

E: Posterior ligament structures dissected off the talus and anterior loosened off the anterior colliculus of the medial malleolus.

F: Orientation of ligamentous reduction at the most common injury pattern.

G: Suture anchor fixed in the anterior colliculus of the medial malleolus. Drilling for the posterior suture anchor in the talus' medial side just inferior to the colliculus posterior of the medial malleolus.

F: Knots tied firmly. We suggest knot-pusher to get to the knot especially for the posterior anchor hiding just inferior to the colliculus posterior of the medial malleolus.
