# Supplementary material for: The Benefit of Repairing the Deltoid Ligament in Unstable Ankle Fractures: Patient-Reported Functional Outcome and Radiological Stability Measurements; a Clinical Trial Protocol
Source: Foot Ankle Orthop. 2025 Nov 12;10(4):24730114251386735. doi: 10.1177/24730114251386735 (PMC12615956; doi:10.1177/24730114251386735)
Supplement: sj-pdf-3-fao-10.1177_24730114251386735 – Supplemental material for The Benefit of Repairing the Deltoid Ligament in Unstable Ankle Fractures: Patient-Reported Functional Outcome and Radiological Stability Measurements; a Clinical Trial Protocol [file sj-pdf-3-fao-10.1177_24730114251386735.pdf]

CONSORT 2010 Flow Diagram

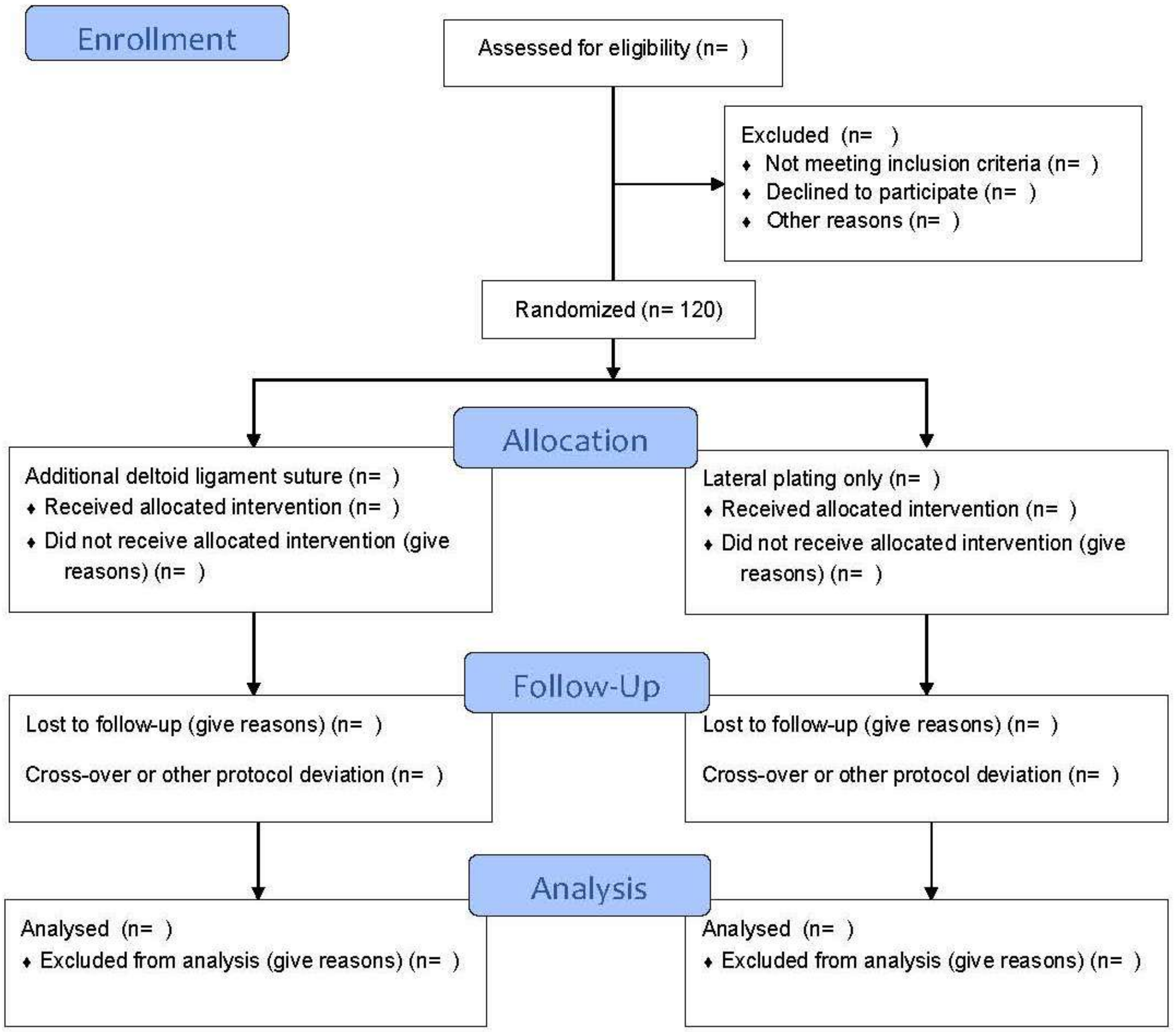

Patients are eligible if they present to one of the participating hospitals and comply with the inclusion and exclusion criteria.	
**Inclusion criteria** 18-65 years of age at presentation	**Exclusion criteria**
Initial medial clear space (MCS) ≥7mm or weightbearing x-ray evaluated as unstable (side to side difference >1 mm ***or***	Assumed not compliant (drug use, cognitive- and/or psychiatric disorders).
Fracture dislocation (*when doubt about state prior to reduction shall WBXR be performed*)	Insufficient language skills (Scandinavian)
Able to walk without aids before the injury	Multi-trauma or pathologic fracture
Posterior malleolus fragment Mason & Molloy 1 or no posterior malleolus fragment[15]	Neuropathies and symptomatic generalized joint disease such as Rheumatoid Arthritis
Surgery planned within 2 weeks after injury and available for follow up	Previous ipsilateral former ankle surgery or fracture or previous injury with marked sequela of the lower limb
No syndesmotic screw or suture button planned prior to the surgical procedure	Open fx Gustilo-Anderson II or more[23] or other medial soft tissue problem considerably increasing risk of additional medial approach to the ankle.
No other more severe condition in the same extremity	
