# Supplementary material for: The Benefit of Repairing the Deltoid Ligament in Unstable Ankle Fractures: Patient-Reported Functional Outcome and Radiological Stability Measurements; a Clinical Trial Protocol
Source: Foot Ankle Orthop. 2025 Nov 12;10(4):24730114251386735. doi: 10.1177/24730114251386735 (PMC12615956; doi:10.1177/24730114251386735)
Supplement: sj-pdf-7-fao-10.1177_24730114251386735 – Supplemental material for The Benefit of Repairing the Deltoid Ligament in Unstable Ankle Fractures: Patient-Reported Functional Outcome and Radiological Stability Measurements; a Clinical Trial Protocol [file sj-pdf-7-fao-10.1177_24730114251386735.pdf]

Region:	Saksbehandler:	E-post:	Telefon:	Vår dato:	Vår referanse:
REK sør-øst B	Camilla Oppegård	rek-sorost@medisin.uio.no	+47 22855240	26.09.2024	496556

Marius Molund

Prosjektsøknad: Bør deltoidligamentet repareres ved ustabile ankelfrakturer?
Søknadsnummer: 496556
Forskningsansvarlig institusjon: Sykehuset Østfold HF
Samarbeidende forskningsansvarlige institusjoner: Sykehuset Levanger HF, Nord-Trøndelag Helseforetak, St. Olavs Hospital HF, Helse Stavanger HF - Stavanger universitetssjukehus, Helse Bergen HF - Haukeland universitetssykehus, Ålesund sjukehus, Sykehuset Innlandet HF

Prosjektsøknad: Endring godkjennes med vilkår

Vi viser til søknad om prosjektendring mottatt 15.06.2024 og supplerende informasjon mottatt 02.07.2024, 04.07.2024 og 13.09.2024 for ovennevnte forskningsprosjekt. Søknaden er behandlet av leder for REK sør-øst B på delegert fullmakt fra komiteen, med hjemmel i forskningsetikkforskriften § 7, første ledd, tredje punktum. Søknaden er vurdert med hjemmel i helseforskningsloven § 11

REKs vurdering

Saksgang
Forhåndsgodkjenning av prosjektet
Prosjektet ble førstegang behandlet i komiteens møte den 21.09.2022. Formålet med studien er å undersøke om reparasjon av ankelens deltoidligament ved operasjon av ankelbrudd bedrer resultatet av den operative behandlingen. Pasientene i studien vil randomiseres til to behandlingsarmer; 1) reparasjon av deltoidligamentet i tillegg til tradisjonell bruddoperasjon, eller 2) kun tradisjonell bruddoperasjon.

Prosjektet ble godkjent med følgende vilkår i vedtak av 13.10.2022:

1. Alle prosjektmedarbeidere må meldes inn.
2. Oppfølgingsperiode og prosjektets sluttdato må være i overensstemmelse, og prosjektperioden lang nok til at den dekker publisering.
3. Det forutsettes at det ikke er behandlende lege som innhenter samtykke til deltakelse, jf. helseforskningsloven § 13, tredje ledd.
4. Manglende spørreskjema må sendes REK til orientering når disse foreligger.
5. Informasjons- og samtykkeskrivet må revideres i henhold til ovenstående merknader.

Endringssøknad den 15.06.2024, samt supplerende informasjon den 02.072024 og 04.07.2024.
De omsøkte endringene i prosjektet er omfatter både endringer som svar på vilkår ved forhåndsgodkjenningen og flere organisatoriske endringer.

Komiteens vurdering av endringen, jf brev den 04.09.2024
Slik REK oppfatter henvendelsen består endringene av:

1. Ny prosjektleder
Ny prosjektleder er overlege Frede Frihagen ved Sykehuset Østfold. CV for ny prosjektleder ble ettersendt den 02.07.2024. REK har ingen forskningsetiske innvendinger til bytte av prosjektleder, og ny prosjektleder vil bli lagt til i saksmappen ved endelig godkjenning av endringssøknaden.

2. Endret prosjektperiode
Det fremkommer av endringssøknaden at prosjektet nå er tildelt forskningsmidler og midler til en stipendiat, slik at prosjektet nå kan starte opp. Oppstart er satt til 01.08.2024 med estimert sluttdato 31.12.2033. Det er fortsatt ikke samsvar i angivelsen av prosjektperiode i søknad og dokumenter. Det fremkommer av samtykkeskrivet at data skal brukes frem til 2032, mens protokollen har en tentativ tidsplan frem til og med 2029. Før REK kan ta stilling til endringssøknaden må det redegjøres for tidsplan og planlagt sluttdato, og det må være samsvar mellom de ulike dokumentene.

3. Samarbeidende institusjoner
Slik REK forstår endringsmeldingen er prosjektet nå et samarbeidsprosjekt mellom Sykehuset Østfold (som koordinerende forskningsansvarlig institusjon) og NTNU (forskningsansvarlig institusjon hvor stipendiat er tatt opp og biveileder arbeider). Det fremkommer av endringssøknaden at det er utarbeidet samarbeidsavtaler mellom Sykehuset Østfold og NTNU om datadeling. NTNU skal være databehandlingsansvarlig institusjon og har også en «databehandleravtale med Helse Midt-Norge IT (Hemit) som databehandler gjennnom "eForsk", en database som kommuniserer med pasientene gjennom HelseNorge om oppfølgingsdata, og som også brukes i registrering av observasjoner fra behandling av brudd ved deltakende sykehus og seinere kontroller med røntgenbilder.»

REK tar redegjørelsen til orientering. Det fremkommer derimot at det også vil være flere sykehus involvert i prosjektet, hvor det også skal rekrutteres deltagere. Det vil lages tilsvarende samarbeidsavtaler med disse sykehusene om datautlevering til NTNU/Hermit. REK etterspurte den 03.07.2024 om en avklaring av hvilken rolle de ulike rekrutterende sykehusene og medarbeidere ved disse skal ha (og hvorvidt disse ville være samarbeidende eller forskningsansvarlige institusjoner. Det er redegjort for rollen til følgende institusjoner, som utover Sykehuset Østfold og NTNU, vil være involvert i prosjektet:

- *Sykehuset Levanger: stipendiatens kliniske arbeidsplass, samt rekruttering og oppfølging av pasienter*
- *Sykehuset Innlandet, Gjøvik og Elverum: samarbeidende institusjon og rekruttering og oppfølging av pasienter*
- *Haukeland Sykehus: samarbeidende institusjon og rekruttering og oppfølging av pasienter*

- *Stavanger Universitetssykehus: samarbeidende institusjon og rekruttering og oppfølging av pasienter*
- *St. Olavs Hospital: samarbeidende institusjon og rekruttering og oppfølging av pasienter*
- *Ålesund sykehus: samarbeidende institusjon og rekruttering og oppfølging av pasienter*

Da det skal rekrutteres pasienter på de ulike sykehusene og det er her pasientoppfølgingen vil foregå, mener REK at disse sykehusene må meldes inn som forskningsanvarlige institusjoner. Dette vil i tillegg medføre at disse institusjonene vil få en kopi av REKs vedtak

4. Endring blant prosjektmedarbeidere
Følgende nye medarbeidere er lagt til i saksmappen:

- *Henning Klasen Hansen, Sykehuset Innlandet HF*
- *Jakup Andreas Thomsen, Ålesund sjukehus*
- *Øystein Bakke Larsen, St. Olavs Hospital HF*
- *Kristian Pilskog, Helse Bergen HF - Haukeland universitetssykehus*
- *Greger Lønne, Norges teknisk-naturvitenskapelige universitet*

REK har ingen forskningsetiske innvendinger, og nye medarbeider vil legges til saksmappen ved endelig godkjenning av endringsmeldingen. Det fremkommer for øvrig at tidligere prosjektleder skal være hovedveileder for stipendiaten og må følgelig legges til som prosjektmedarbeider. I tillegg er det oppgitt medarbeidere i revidert protokoll og som ikke er meldt inn som medarbeidere i portalen. Alle medarbeidere som skal bidra vitenskapelig i studien, må meldes inn.

5. Endringer i protokoll
Det er gjort relativt store endringer i protokollen. Utover at prosjektet skal rekruttere pasienter fra flere sykehus, er prosjektet delt opp i flere arbeidspakker. REK ba den 03.07.2024 om en beskrivelse og begrunnelse for hvilke vesentlige endringene som er gjort i protokollen. Prosjektleder sendte supplerende informasjon den 04.07.2024. Her fremheves følgende hoved-endringer:

For arbeidspakke 1, tilsvarer i hovedsak tidligere protokoll med følgende endringer:

- *Etter nye styrkeberegninger er antall pasienter som skal inkluderes redusert fra 160 til 120.*
- *Endring blant spørreskjema for sekundært endepunkter: MOxFQ er byttet ut med PROMIS*
- *Oppfølgingstidspunktet er endret fra 6 måneder til 12 uker/3 måneder. CT-underøskelse er tatt ut*
- *Lagt til en oppfølging etter 5 år med røntgen*
- *Lagt til røntgenbilde-spesialopptak som vurderer stabilitet tilsvarende ankelens leddbånd på innsida kalt gravity test-bilder til taking på 1 og 5-årskontroll*
- *eForsk er tatt inn som elektronisk løsning for både kommunikasjon med pasienter for elektronisk innhenting av opplysninger/svar på spørreskjema og datalagring*

Arbeidspakke 2:

- *ny arbeidspakke som inkluderer analyse av røntgenbildene (WBXR eller gravity test) for sammenligning av stabilitet og artrose*

Arbeidspakke 3:

- *Ny arbeidspakke som i hovedsak omfatter en valideringsstudie av spørreskjema (Ankle Fracture Outcome of Reabhilitation Measure, A-FORM) som er oversatt til norsk. Skjemaet skal besvares 2 ganger ca 1 år etter operasjon.*

REK har ingen forskningsetiske innvendinger til de beskrevne endringene i protokoll. Slik vi forstår vil arbeidspakke 3 i tillegg til å være en valideringsstudie, også bruker PROM data til å skaffe ny kunnskap om helse i prosjektet, slik at disse tre arbeidspakkene er tett koblet sammen. Etter komiteens vurdering faller dermed alle de tre arbeidspakkene av REKs forhåndgodkjenning. Spørreskjemaene (A-FORM, PROMIS og VAS) som skal benyttes må sendes REK til orientering

6. Revidert informasjons- og samtykkeskriv
Det er lagt ved et revidert informasjons- og samtykkeskriv hvor hele skrivet er sporet med endringer. Prosjektleder skriver «Vedrørende pasientinformajsonsskrivet er hovedendringen at deltakersteder samt kontaktinfo er oppdatert. Etter anbefaling fra brukerrepresentant ved sykehuset Østfold er opplysningene som pasienten finner viktigst i første omgang samlet på første side og det er gjort forsøk på språklige forenklinger. eForsk er beskrevet.»

REK har noen innvendinger til samtykkeskrivet som må revideres før dette kan tas i bruk:

1. *Under avsnittet «Mulige fordeler og ulemper» må følgende setning tas ut: «Ved å delta i prosjektet vil du få en tettere og mer helhetlig oppfølging.»*
2. *Under avsnittet «Hva skje med opplysningen om deg» må «anonymiserte» erstattes med avidentifiserte», og det må være samsvar mellom hvor lenge data skal benyttes og prosjektperioden som er oppgitt i andre dokumenter.*

Annet
REK viser til at prosjektet ble godkjent med vilkår. Det er i tilbakemeldingen ikke eksplisitt svart ut for disse, men endringen berører til dels vilkårene 1, 2 4, og 5. Som REK har påpekt ovenfor er det fortsatt uklarheter knyttet til både medarbeidere som er innmeldt, prosjektperioden, manglende spørreskjema og justeringer i samtykkeskriv. Når det gjelder vilkår 3, er det i revidert protokoll ikke beskrevet hvordan pasientene skal rekrutteres. REK gjør igjen oppmerksom på at iht. helseforskningsloven § 13, tredje ledd, så skal ikke samtykke innhentes av en som pasienten kan sies å være i et avhengighetsforhold til, slik som behandlende lege. Det må beskrives en rekrutteringsprosedyre i protokollen.

Konklusjon
Før REK kan ta endelig stilling til endringssøknaden må

1. *Det må redegjøres for tidsplan og planlagt sluttdato, og det må være samsvar mellom de ulike protokoll, samtykkeskriv og omsøkte prosjektperiode.*
2. *Sykehus hvor pasienter skal rekrutteres og følges opp, må meldes inn som forskningsanvarlige institusjoner (Sykehuset i Østfold vil være koordinerende forskningsansvarlig).*

3. *Alle medarbeidere som skal bidra vitenskapelig i studien, må meldes inn.*
4. *Spørreskjemaene (A-FORM, PROMIS og VAS) som skal benyttes må sendes REK til orientering.*
5. *Informasjons- og samtykkeskrivet må revideres i henhold til komiteens merknader.*
6. *Det må i revidert protokoll beskrives en rekrutteringsprosedyre som er i henhold til helseforskningsloven § 13 (samtykke skal ikke innhentes av en som pasienten kan sies å være i et avhengighetsforhold til, slik som behandlende lege).*

Tilbakemelding fra prosjektleder 13.09.2024
Prosjektleder sendte svar på komiteens merknader den 13.09.2024. Vedlagt tilbakemeldingen var:

- Oppdatert protokoll
- Informasjonsskriv med samtykkeskjema (datert 1.1 september 2024)
- A-FORM og VAS Pain
- PROMIS Mobility Bank 2.1

Komiteens tilleggsvurdering
Det er i tilbakemeldingen redegjort for de merknadene komiteen hadde. Når det gjelder merknaden knyttet til punkt 2 og 3 (andre forskningsansvarlige institusjoner og nye medarbeidere) er dette meldt inn som en egen endringsmelding (mottatt 13.09.2024), og disse to punktene vil svares ut i eget vedtak. Nedenfor følger komiteens vurdering av de øvrige punktene:

<u>Vedrørende punkt 1 – tidsplan og planlagt sluttdato</u>
Det er redegjort for og beskrevet en tentativ fremdriftsplan (inkludert 5 års oppfølging av pasientene og analyser/publikasjoner, med estimert sluttdato 31.12.2033, i protokollen. Dette er også i samsvar med informasjon i samtykkeskrivet. REK tar redegjørelsen til orientering.

<u>Vedrørende punkt 4 – spørreskjema</u>
Vedlagt tilbakemeldingen var det lagt ved norske oversettelser av A-FORM, VAS, samt en engelsk versjon av PROMIS). Når det gjelder PROMIS skriver prosjektleder at «PROMIS mobility bank 2.1. (vedlagt) inneholder veldig mange spørsmål. Denne vil bli brukt først ved ett års oppfølging. Da med et utvalg av spørsmålene som ennå ikke er bestemt, forhåpentlig vil vi ha en løsning klar med computer adaptive testing (CAT) satt opp i eForsk der svar på innledende spørsmål betinger hva slags oppfølgende spørsmål som blir brukt. Oppdatering skal komme om dette.» REK tar redegjørelsen til orientering og ber om at det sendes en endringsmelding med oppdatert spørreskjema (på norsk) når det er tatt stilling til hvilke utvalg med spørsmål som blir benyttet ved 1 års oppfølging.

<u>Vedrørende punkt 5 – informasjons- og samtykkeskriv</u>
Vedlagt tilbakemeldingen var et revidert skriv som ivaretar de merknaden komiteen hadde. Beskrivelsen av pasientenes oppfølging beskrives på en nøktern måte, det er oppgitt tentativ prosjetkslutt i 2033 og at data-analysene vil være på et avidentifisert datasett.

<u>Vedrørende punkt 6 – rekrutteringsprosedyre</u>
I revidert protokoll er det lagt til utfyllende informasjon om rekrutteringsprosedyren. Her presiseres det at deltager vil forespørres og samtykke vil innhentes av andre enn behandlende kirurg. REK har ingen innvendinger til rekrutteringsprosedyren slik den nå er

beskrevet. REK legger til grunn at det gis tilstrekkelig tid mellom informasjon om studien gis til et eventuelt samtykke innhentes, slik at deltagerne sikres tilstrekkelig betekningstid.

Konklusjon
Samlet er det komiteens vurdering at prosjektendringen kan godkjennes. Det stilles som vilkår at det sendes en ny endringsmelding med oppdatert spørreskjema (PROMIS på norsk) når det er tatt stilling til hvilke utvalg med spørsmål som skal benyttes ved 1 års oppfølging.

Vedtak
REK har gjort en forskningsetisk vurdering av endringene i prosjektet og godkjenner prosjektet slik det nå foreligger, jf. helseforskningsloven § 11.

Prosjektet er godkjent frem til 31.12.2033. Etter prosjektslutt skal opplysningene oppbevares i fem år for dokumentasjonshensyn. Enhver tilgang til prosjektdataene skal da være knyttet til behovet for etterkontroll. Prosjektdata skal således ikke være tilgjengelig for prosjektet. Prosjektleder og forskningsansvarlig institusjon er ansvarlig for at opplysningene oppbevares indirekte personidentifiserbart i denne perioden, dvs. atskilt i en nøkkel- og en datafil. Etter disse fem årene skal data slettes eller anonymiseres. Vi gjør oppmerksom på at anonymisering kan være mer omfattende enn å kun slette koblingsnøkkelen, jf. Datatilsynets veileder om anonymiserings-teknikker.

Tillatelsen er gitt under forutsetning av at prosjektendringen gjennomføres slik det er beskrevet i prosjektendringsmeldingen og endringsprotokoll, og i henhold til de bestemmelser som følger av helseforskningsloven med forskrifter.

Sluttmelding
Prosjektleder skal sende sluttmelding til REK på eget skjema via REK-portalen senest 6 måneder etter sluttdato 31.12.2033, jf. helseforskningsloven § 12. Dersom prosjektet ikke starter opp eller gjennomføres meldes dette også via skjemaet for sluttmelding.

Søknad om endring
Dersom man ønsker å foreta vesentlige endringer i formål, metode, tidsløp eller organisering må prosjektleder sende søknad om endring via portalen på eget skjema til REK, jf. helseforskningsloven § 11.

Klageadgang
Du kan klage på REKs vedtak, jf. forvaltningsloven § 28 flg. Klagen sendes på eget skjema via REK portalen. Klagefristen er tre uker fra du mottar dette brevet. Dersom REK opprettholder vedtaket, sender REK klagen videre til Den nasjonale forskningsetiske komité for medisin og helsefag (NEM) for endelig vurdering, jf. forskningsetikkloven § 10 og helseforskningsloven § 10.

Med vennlig hilsen

Ragnhild Emblem

Professor, dr. med.
leder REK sør-øst B

Camilla Oppegård
Seniorrådgiver
REK sør-øst

Kopi til:

Sykehuset Østfold HF
Frede Frihagen
Sykehuset Levanger HF, Nord-Trøndelag Helseforetak, St. Olavs Hospital HF, Helse Stavanger HF - Stavanger universitetssjukehus, Helse Bergen HF - Haukeland universitetssykehus, Ålesund sjukehus, Sykehuset Innlandet HF

Region:	Saksbehandler:	Telefon:	Vår dato:	Vår referanse:
REK sør-øst B	Marianne Bjørnerem	22845531	13.10.2022	496556

Marius Molund

Prosjektsøknad: Bør deltoidligamentet repareres ved ustabile ankelfrakturer?
Søknadsnummer: 496556
Forskningsansvarlig institusjon: Sykehuset Østfold HF
Samarbeidende forskningsansvarlige institusjoner: Sykehuset Innlandet HF

Prosjektsøknad godkjennes med vilkår

Søkers beskrivelse

Formålet med prosjektet er å prøve å bedre operativ behandling av ankelbrudd.
Det er tilkommet ganske mye ny viten om behandling av ankelbrudd i seinere år. Dette har medført ressursbesparing ved at færre ankelbrudd behøver operasjon.

Imidlertid er de ankelbruddene som nå opereres mer ustabile/mer omfattende skader enn ankelbruddene som ble operert tidligere. Forventelig er resultatet innafor gruppa av ankelbrudd som opereres nå i dag ikke så bra som for gjennomsnittet for ankelbrudd som ble operert tidligere, der det var mildere skader som egentlig ikke trenger operasjon. Vi antar at resultatet av behandling av disse skadene kan bli bedre med en mer stabil reparasjon. Det er påvist at det er sentralt om det dype deltoidligamentet på innsida av ankelen er avrevet eller ikke. Kadaverstudier har vist at det å reparere dette ligamentet i tillegg til å reparere selve beinbruddet med plate og skruestabilisering, gir en langt bedre stabilitet. Vi tror dette kan gi mulighet for et bedre resultat.

Vi vil inkludere pasienter som har brudd i nederst i leggbeinet (på utsida av ankelen); laterale malleol alene, der ankelen har vært ute av ledd ved bruddet eller der røntgenutredning viser at skaden er ustabil. Vi vil trekke lodd mellom tradisjonell behandling med reparasjon med skrue og plate alene i den ene gruppa og reparasjon av leddbåndet på innsida også i den andre. Vi vil følge pasientene med pasientrapporterte mål for smerte og funksjon (PROMs) der Olerud-Molander er hovedmål for resultatet. Vi ønsker å samle 120 pasienter. De vil bli kontrollert på vanlig måte med røntgen for å se på stilling av brudd og eventuell slitasjegiktutvikling på 6 uker og 6 mnd, og ved 1 og 2 år og rg på 5 år, også med PROMs på nevnte tidspunkter

Vi viser til søknad om forhåndsgodkjenning av ovennevnte forskningsprosjekt. Søknaden ble behandlet av Regional komité for medisinsk og helsefaglig forskningsetikk (REK sør-øst B) i møtet 21.09.2022. Vurderingen er gjort med hjemmel i helseforskningslovens § 10, jf. forskningsetikkloven § 10.

REKs vurdering

Formålet med prosjektet, slik komiteen forstår søknad og protokoll, er å undersøke om reparasjon av ankelens deltoidligament ved operasjon av ankelbrudd bedrer resultatet av den operative behandlingen.

Prosjektet er randomisert med to behandlingsarmer; reparasjon av deltoidligamentet i tillegg til tradisjonell bruddoperasjon, eller kun tradisjonell bruddoperasjon.

Prosjektgruppe, oppfølging og prosjektperiode

Det fremgår av forskningsprotokollen at det er flere prosjektmedarbeidere enn de som er listet i søknaden. Det bes om at alle prosjektmedarbeidere meldes inn til REK, det må oppgis navn, akademisk grad, stilling, tilhørende institusjon og prosjektrolle.

Prosjektet har omsøkt sluttdato 31.12.2027. Deltakere vil ifølge forskningsprotokollen følges opp etter 6 uker, 6 måneder, 1, 2 og 5 år. Det fremkommer imidlertid av informasjons- og samtykkeskrivet at prosjektet er planlagt å vare til 2028, og at det er planlagt oppfølging i to år.

REK minner om at prosjektslutt er den datoen der datainnsamling, analysering og publisering er ferdigstilt. Prosjektet må ha en gyldig prosjektperiode for all behandling av studiedata og personopplysninger. Dersom deltakere skal følges opp i fem år, vil det være behov for lengre prosjektperiode. Det bes derfor om en redegjørelse for tidsplan og planlagt sluttdato, og at denne tar høyde for at prosjektet skal være fullstendig ferdigstilt innen denne datoen.

Studiepopulasjon og rekruttering

Det er planlagt inklusjon av totalt 120 deltakere.

Potensielle deltakere forespørres om deltakelse ved innleggelse på sykehuset av journalskrivende lege. Samtykke vil avgis til ansvarlig sykepleier eller vakthavende eller visittgående lege.

Komiteen gjør oppmerksom på at iht. helseforskningsloven § 13, tredje ledd, så skal ikke samtykke innhentes av en som pasienten kan sies å være i et avhengighetsforhold til, slik som behandlende lege.

Studiedata

Det skal innsamles studiedata i form av

- Spørreskjema (PROMS; Olerud-Molander, Manchester Oxford Foot and ANkle Questionnaire (MOxFQ), A-FORM, SEFAS, EQ5D-5L, VAS pain)
- Observasjoner med opptak (røntgenbilder)
- Kliniske undersøkelser (Kontroller som vanlig ved 6 uker og 6 mnd med rtg og klinisk undersøkelse)

A-FORM og VAS pain lå ikke vedlagt søknaden. Det informeres i søknaden om at A-FORM er i en prosess med oversettelse og validering på norsk. Det bes om at skjemaene sendes REK til orientering når disse foreligger i endelig versjon.

Informasjons- og samtykkeskriv

Komiteen har noen merknader til det vedlagte skrivet.

- Sluttdato og oppfølging av deltakere må revideres i henhold til ovenstående merknad.
- Haanæs oppgis som prosjektleder. Dette må endres til Molund, i henhold til søknaden.

Konklusjon

Komiteen anser prosjektet som nyttig og godt utformet, og godkjenner søknaden med følgende vilkår:

1. Alle prosjektmedarbeidere må meldes inn.
2. Oppfølgingsperiode og prosjektets sluttdato må være i overensstemmelse, og prosjektperioden lang nok til at den dekker publisering.
3. Det forutsettes at det ikke er behandlende lege som innhenter samtykke til deltakelse, jf. helseforskningsloven § 13, tredje ledd.
4. Manglende spørreskjema må sendes REK til orientering når disse foreligger.
5. Informasjons- og samtykkeskrivet må revideres i henhold til ovenstående merknader.

Reviderte dokumenter sendes inn med sporede endringer som viser hva som har blitt endret, lagt til og/eller slettet, samt merket med oppdatert versjonsnummer og dato. Svar på vilkår sendes inn via skjemaet «Endring og /eller henvendelse» i REK portalen.

Vedtak

REK har gjort en helhetlig forskningsetisk vurdering av alle prosjektets sider. Prosjektet godkjennes med hjemmel i helseforskningsloven § 10, under forutsetning av at ovennevnte vilkår oppfylles.

I tillegg til vilkår som fremgår av dette vedtaket, er godkjenningen gitt under forutsetning av at prosjektet gjennomføres slik det er beskrevet i søknad og protokoll, og de bestemmelser som følger av helseforskningsloven med forskrifter.

Komiteens avgjørelse var enstemmig.

Prosjektet er godkjent frem til 31.12.2027.

Etter prosjektslutt skal opplysningene oppbevares i fem år for dokumentasjonshensyn. Enhver tilgang til prosjektdataene skal da være knyttet til behovet for etterkontroll. Prosjektdata skal således ikke være tilgjengelig for prosjektet. Prosjektleder og forskningsansvarlig institusjon er ansvarlig for at opplysningene oppbevares indirekte personidentifiserbart i denne perioden, dvs. atskilt i en nøkkel- og en datafil.

Etter disse fem årene skal data slettes eller anonymiseres. Vi gjør oppmerksom på at anonymisering kan være mer omfattende enn å kun slette koblingsnøkkelen, jf. Datatilsynets veileder om anonymiserings-teknikker.

Vi gjør samtidig oppmerksom på at det også må foreligge et behandlingsgrunnlag etter personvernforordningen. Dette må forankres i egen institusjon.

Sluttmelding
Prosjektleder skal sende sluttmelding til REK på eget skjema via REK-portalen senest 6 måneder etter sluttdato 31.12.2027, jf. helseforskningsloven § 12. Dersom prosjektet ikke starter opp eller gjennomføres meldes dette også via skjemaet for sluttmelding.

Søknad om endring
Dersom man ønsker å foreta vesentlige endringer i formål, metode, tidsløp eller organisering må prosjektleder sende søknad om endring via portalen på eget skjema til REK, jf. helseforskningsloven § 11.

Klageadgang
Du kan klage på REKs vedtak, jf. forvaltningsloven § 28 flg. Klagen sendes på eget skjema via REK portalen. Klagefristen er tre uker fra du mottar dette brevet. Dersom REK opprettholder vedtaket, sender REK klagen videre til Den nasjonale forskningsetiske komité for medisin og helsefag (NEM) for endelig vurdering, jf. forskningsetikkloven § 10 og helseforskningsloven § 10.

Med vennlig hilsen

Ragnhild Emblem
Professor, dr. med.
Leder REK sør-øst B

Marianne Bjørnerem
Rådgiver, REK sør-øst

Kopi til:

Sykehuset Østfold HF
Sykehuset Innlandet HF

Region:	Saksbehandler:	E-post:	Telefon:	Vår dato:	Vår referanse:
REK sør-øst B	Camilla Oppegård	rek-sorost@medisin.uio.no	+47 22855240	23.01.2025	496556

Frede Frihagen

Prosjektsøknad: Bør deltoidligamentet repareres ved ustabile ankelfrakturer?
Søknadsnummer: 496556
Forskningsansvarlig institusjon: Sykehuset Østfold HF
Samarbeidende forskningsansvarlige institusjoner: Oslo universitetssykehus HF, Sykehuset Levanger HF, Nord-Trøndelag Helseforetak, Nordlandssykehuset HF, St. Olavs Hospital HF, Helse Stavanger HF - Stavanger universitetssjukehus, Helse Bergen HF - Haukeland universitetssykehus, Ålesund sjukehus, Sykehuset Innlandet HF

Prosjektsøknad: Endring godkjennes

Vi viser til søknad om prosjektendring mottatt 20.12.2024 og supplerende informasjon mottatt 23.01.2025 for ovennevnte forskningsprosjekt. Søknaden er behandlet av sekretariatet i Regional komité for medisinsk og helsefaglig forskningsetikk (REK) sør-øst B på delegert fullmakt fra komiteen, med hjemmel i forskningsetikkforskriften § 7, første ledd, tredje punktum. Søknaden er vurdert med hjemmel i helseforskningsloven § 11.

REKs vurdering
Endringene omfatter:

1. Nye forskningsansvarlige institusjoner
Studien er en multisenterstudie med Sykehuset i Østfold som koordinerende forskningsansvarlig institusjon.

Det søkes om å legge til to ny forskningsansvarlige institusjoner: OUS - Ullevål og Nordlandssykehuset. Begge institusjonene skal bidra i rekruttering og pasientoppføling.

Listen over andre norske forskningsansvarlige institusjoner (med kontaktpersoner) er for øvrig oppdatert i samsvar tidligere oversikt i protokollen. Protokollen er ellers oppdatert med de to nye institusjonene.

REK har ingen forskningsetiske innvendinger, og listen over forskningsansvarlige institusjoner er oppdatert i saksmappen.

2. Revidert informasjons- og samtykkeskriv
Skrivet er oppdatert med kontaktinformasjon til personvernombud ved de nye institusjonene og logo til sykehusene som deltar i studien. REK tar endringene til orientering.

Vedtak
REK har gjort en forskningsetisk vurdering av endringene i prosjektet, og godkjenner prosjektet slik det nå foreligger, jf. helseforskningsloven § 11.

Tillatelsen er gitt under forutsetning av at prosjektet gjennomføres slik det er beskrevet i søknaden, endringssøknad, oppdatert protokoll og de bestemmelser som følger av helseforskningsloven med forskrifter.

Sluttmelding
Prosjektleder skal sende sluttmelding til REK på eget skjema via REK-portalen senest 6 måneder etter sluttdato 31.12.2033, jf. helseforskningsloven § 12. Dersom prosjektet ikke starter opp eller gjennomføres meldes dette også via skjemaet for sluttmelding.

Søknad om endring
Dersom man ønsker å foreta vesentlige endringer i formål, metode, tidsløp eller organisering må prosjektleder sende søknad om endring via portalen på eget skjema til REK, jf. helseforskningsloven § 11.

Klageadgang
Du kan klage på REKs vedtak, jf. forvaltningsloven § 28 flg. Klagen sendes på eget skjema via REK portalen. Klagefristen er tre uker fra du mottar dette brevet. Dersom REK opprettholder vedtaket, sender REK klagen videre til Den nasjonale forskningsetiske komité for medisin og helsefag (NEM) for endelig vurdering, jf. forskningsetikkloven § 10 og helseforskningsloven § 10.

Med vennlig hilsen

Jacob Hølen
Direktør REK sør-øst/KULMU

Camilla Oppegård
Seniorrådgiver, REK sør-øst

Kopi til:

Sykehuset Østfold HF
Oslo universitetssykehus HF, Sykehuset Levanger HF, Nord-Trøndelag Helseforetak, Nordlandssykehuset HF, St. Olavs Hospital HF, Helse Stavanger HF - Stavanger universitetssjukehus, Helse Bergen HF - Haukeland universitetssykehus, Ålesund sjukehus, Sykehuset Innlandet HF
Marius Molund, Esten Konstad Konstad Øiaas Haanæs
